# Antibody-drug conjugates in HER2-positive advanced or metastatic gastric cancer: a systematic review and meta-analysis

**DOI:** 10.3389/fonc.2025.1684873

**Published:** 2025-10-29

**Authors:** Jiayang Li, Shuangyu Chen, Yinying Chai, Shengliang Qiu

**Affiliations:** ^1^ The First Clinical Medical School of Zhejiang Chinese Medical University, Hangzhou, Zhejiang, China; ^2^ The First Affiliated Hospital of Zhejiang Chinese Medical University (Zhejiang Provincial Hospital of Chinese Medicine), Hangzhou, Zhejiang, China

**Keywords:** antibody-drug conjugate, HER2-positive, advanced gastric cancer, metastatic gastric cancer, subsequent-line treatments, meta-analysis, efficacy, safety

## Abstract

**Background:**

Antibody-drug conjugates (ADCs) are an emerging therapy for HER2-positive advanced gastric cancer (AGC), yet their comparative efficacy and safety remain unclear. This systematic review and meta-analysis aimed to evaluate the clinical outcomes of different ADCs in this patient population.

**Methods:**

A systematic search of PubMed, Embase, Cochrane, and Scopus databases was performed to identify relevant studies. The primary endpoint was the pooled overall response rate (ORR), analyzed using a random-effects model. Safety, subgroup analyses, and publication bias were also assessed.

**Results:**

Twelve studies comprising 1041 patients were included. The pooled ORR across all ADCs was 33.4% (95% CI, 26.3%–41.3%). Efficacy varied substantially among agents: trastuzumab deruxtecan (T-DXd) and DP303c demonstrated the highest ORRs (42.5% and 42.9%, respectively), whereas others, such as Trastuzumab emtansine (T-DM1), showed lower efficacy (20.6%). ORR was not significantly affected by prior treatment lines (P = 0.6559) or cohort type (P = 0.7185). The most common adverse events included nausea (47.7%), with grade ≥3 anemia (21.1%) and neutropenia (15.1%) being the most frequent severe toxicities.

**Conclusions:**

The efficacy of ADCs in HER2-positive AGC is highly variable. T-DXd and DP303c appear to be the most active agents, underscoring the critical importance of specific drug selection. Managing toxicities such as anemia and neutropenia is essential for optimizing treatment.

**Systematic review registration:**

https://www.crd.york.ac.uk/PROSPERO/view/CRD420250653886, identifier PROSPERO CRD420250653886.

## 1 Introduction

Gastric cancer (GC) remains a significant global health burden, with advanced or metastatic disease carrying a particularly poor prognosis (1, 2). Within this challenging landscape, the identification of molecular subtypes has been pivotal for developing targeted therapies, and one of the most clinically significant is Human Epidermal Growth Factor Receptor 2 (HER2)-positive disease (3). HER2-positive status, a clinically significant molecular subtype, is present in approximately 7.3–20.2% of advanced GC (4–7). This subtype is associated with aggressive tumor biology and poor prognoses, yields a 5-year survival rate of only 5–20% (8), particularly in the advanced or metastatic setting (9). Common sites for metastasis include lymph nodes, liver, and peritoneum. Patients with advanced disease may present with poor performance status and ascites (10). Consequently, targeting the HER2 pathway has become a cornerstone of therapy for this patient population. For over a decade, the first-line standard of care has been trastuzumab combined with platinum-fluoropyrimidine chemotherapy, marked a pivotal advancement, establishing the first-line standard of care for these patients for over a decade (11, 12). However, its efficacy is often limited by primary or acquired resistance, and the challenge of HER2 expression heterogeneity, including its potential loss post-therapy (13). These limitations have spurred the development of novel HER2-targeted strategies to improve patient outcomes.

To overcome the limitations of traditional HER2 blockade, Antibody-Drug Conjugates (ADCs) have recently emerged as a transformative therapeutic modality for HER2-positive metastatic GC, offering a novel mechanism to deliver potent cytotoxicity directly to cancer cells (11, 14). As a therapeutic modality, ADCs consist of a HER2-targeting antibody linked to a potent cytotoxic payload via a specialized linker (15–17). Upon binding to HER2 and internalization, the payload is released, inducing cancer cell death (18). Furthermore, many modern ADCs feature a “bystander effect,” whereby the membrane-permeable payload can diffuse into adjacent HER2-low or -negative tumor cells, thereby addressing the challenge of tumor heterogeneity (11, 19). This mechanism thereby helps mitigate innate or acquired resistance to HER2-targeted agents. Pivotal trials demonstrated significant improvements in objective response rates (ORR) and survival with trastuzumab deruxtecan (T-DXd) in patients progressing on prior trastuzumab-containing regimens (20, 21). Furthermore, T-DXd’s observed activity in HER2-low GC broadens the scope of HER2-targeted therapy (13, 14).

The evolving landscape and the compelling efficacy shown by various ADCs. However, the rapid development of multiple effective ADCs has outpaced the generation of comparative evidence. The absence of direct head-to-head randomized trials comparing these novel agents hinders evidence-based treatment selection in the second-line setting and beyond (22). Concurrently, their real-world utility is constrained by dose-limiting toxicities (e.g., interstitial lung disease and hematologic events), necessitating safety optimization strategies (23, 24).

Therefore, we conducted this systematic review and meta-analysis to evaluate the clinical efficacy and safety of ADC monotherapy in patients with advanced or metastatic HER2-positive GC, with a focus on the ORR and overall safety profile. This provides a robust statistical approach for synthesizing direct and indirect evidence from individual trials.

## 2 Method

### 2.1 Search strategy

Two investigators independently searched the PubMed, Embase, Cochrane, and Scopus databases. Additional records were identified by screening other sources, including ClinicalTrials.gov, conference proceedings of the American Society of Clinical Oncology (ASCO) and the European Society for Medical Oncology (ESMO), and the reference lists of books and review articles to ensure comprehensive identification of all eligible studies. The literature search was performed without restrictions on publication language, country, region, or ethnicity, and covered the period from the inception of each database to our final search date of June 24, 2025. The search strategy was constructed using a combination of controlled vocabulary terms (e.g., Medical Subject Headings [MeSH] and Emtree) and free-text keywords. These terms were conceptually grouped into four key domains and linked with Boolean operators: (1) gastric cancer (e.g., “gastric neoplasm”), (2) HER2-positive status (e.g., “HER2-positive”, “epidermal growth factor receptor 2”), (3) advanced or metastatic disease (e.g., “advanced cancer”, “metastatic cancer”), and (4) antibody-drug conjugates (e.g., “antibody-drug conjugate”, “Trastuzumab deruxtecan”). A filter for clinical trials was applied where appropriate. The full, detailed search strategy for each database is documented in **Supplementary Methods 1–4**. This systematic review was conducted in accordance with a pre-specified protocol, which was registered with the International Prospective Register of Systematic Reviews (PROSPERO) on February 24, 2025 (Registration No. CRD420250653886). The complete protocol can be accessed at https://www.crd.york.ac.uk/PROSPERO/view/CRD420250653886.

### 2.2 Inclusion and exclusion criteria

Inclusion criteria were as follows: (1) Clinical trials, including randomized controlled trials (RCTs) and single-arm studies; (2) Studies enrolling patients with histologically confirmed HER2-positive locally advanced or metastatic GC; (3) Studies administering ADC monotherapy. Exclusion criteria were as follows: (1) Non-human (animal or *in vitro*) studies; (2) Secondary publications (letters, reviews, meta-analyses, commentaries, case reports, conference abstracts, editorials, expert opinions); (3) Duplicate publications. Two investigators independently established the criteria. A third reviewer adjudicated discrepancies to achieve consensus when required.

### 2.3 Quality assessment

Two reviewers (Li and Chen) independently assessed the risk of bias for all included studies. Any disagreements were resolved by consensus or, if necessary, through arbitration by a third reviewer (Chai). For non-randomized studies, the Risk of Bias in Non-randomized Studies – of Interventions (ROBINS-I) tool was utilized. The overall risk of bias was judged as ‘Low’, ‘Moderate’, ‘Serious’, or ‘Critical’ following evaluation across seven domains: confounding, selection of participants, classification of interventions, deviations from intended interventions, missing data, measurement of outcomes, and selection of the reported result. For randomized controlled trials (RCTs), the Cochrane Risk of Bias 2 (RoB 2) tool was employed using RevMan software (v5.4.1, The Cochrane Collaboration). Guided by standardized signaling questions, each of the five domains—bias arising from the randomization process, deviations from intended interventions, missing outcome data, measurement of the outcome, and selection of the reported result—was categorized as having a ‘Low risk of bias’, ‘Some concerns’, or ‘High risk of bias’. If the meta-analysis included ≥10 studies, potential publication bias for the primary outcome (ORR) was evaluated through visual inspection of funnel plot asymmetry, supplemented by the Begg and Harbord statistical tests.

### 2.4 Data extraction

Two authors (Li and Chen) independently conducted the study selection and data extraction. This process involved a two-stage screening of titles and abstracts, followed by a full-text review of potentially eligible articles. Any discrepancies were resolved by consensus or third-party adjudication. Data were extracted across three domains: (1) Study characteristics: bibliographic details, design, patient baseline data (e.g., age, HER2 status, prior treatments), and the specific ADC regimen. (2) Efficacy outcomes, evaluated per RECIST v1.1: the ORR and the number of patients with a complete response (CR), partial response (PR), stable disease (SD), or progressive disease (PD). (3) Safety profile: the incidence of all-grade and grade ≥3 adverse events (AEs), rates of treatment discontinuation due to AEs, and treatment-related mortality.

### 2.5 Statistical analysis

Data from the included randomized controlled trials (RCTs) were synthesized using Review Manager (RevMan, v5.4.1). All subsequent meta-analyses were conducted with the meta package in R (v4.3.2). Effect sizes were pooled as Risk Ratios (RR) with 95% confidence intervals (CI) for RCTs, and as Proportions with 95% CIs for the single-arm studies.

Inter-study heterogeneity was quantified using the DerSimonian-Laird estimator for tau², Cochran’s Q test (significance threshold: P<0.10) and the I² statistic. A random-effects model was applied when significant heterogeneity (I²>50%) was present; otherwise, a fixed-effect model was employed. Proportions were transformed using logit function to stabilize variances. Clopper-Pearson exact confidence intervals (CIs) were computed for study-specific proportions. Pre-specified subgroup analyses were performed to explore potential sources of heterogeneity. A leave-one-out sensitivity analysis involving all studies was conducted to assess the robustness of the pooled estimates. Publication bias was evaluated by examining funnel plot asymmetry, which was then formally tested using Begg’s rank correlation test and Harbord’s modified regression test to ensure methodological rigor.

### 2.6 Subgroup analysis

Subgroup analyses by: (i) GC patients enrollment proportion (GC-dedicated cohorts versus cohort*s without independent GC data*); (ii) Prior therapeutic lines (low-intensity versus high-intensity). (iii) Drug of intervention (contrast between different drugs). According to oncological conventions, based on their shared biology and treatment paradigms, GC and gastroesophageal junction (GEJ) cancers are combined as ‘gastric cancer’ for subgroup classification. Studies exclusively enrolling either GC or GEJ cancer patients were categorized into the GC-dedicated cohort. Cohorts were stratified by prior therapy lines into low-intensity (median ≤2) and high-intensity subgroups (median ≥3).

## 3 Result

### 3.1 Literature search and baseline characteristics

**Figure 1:**
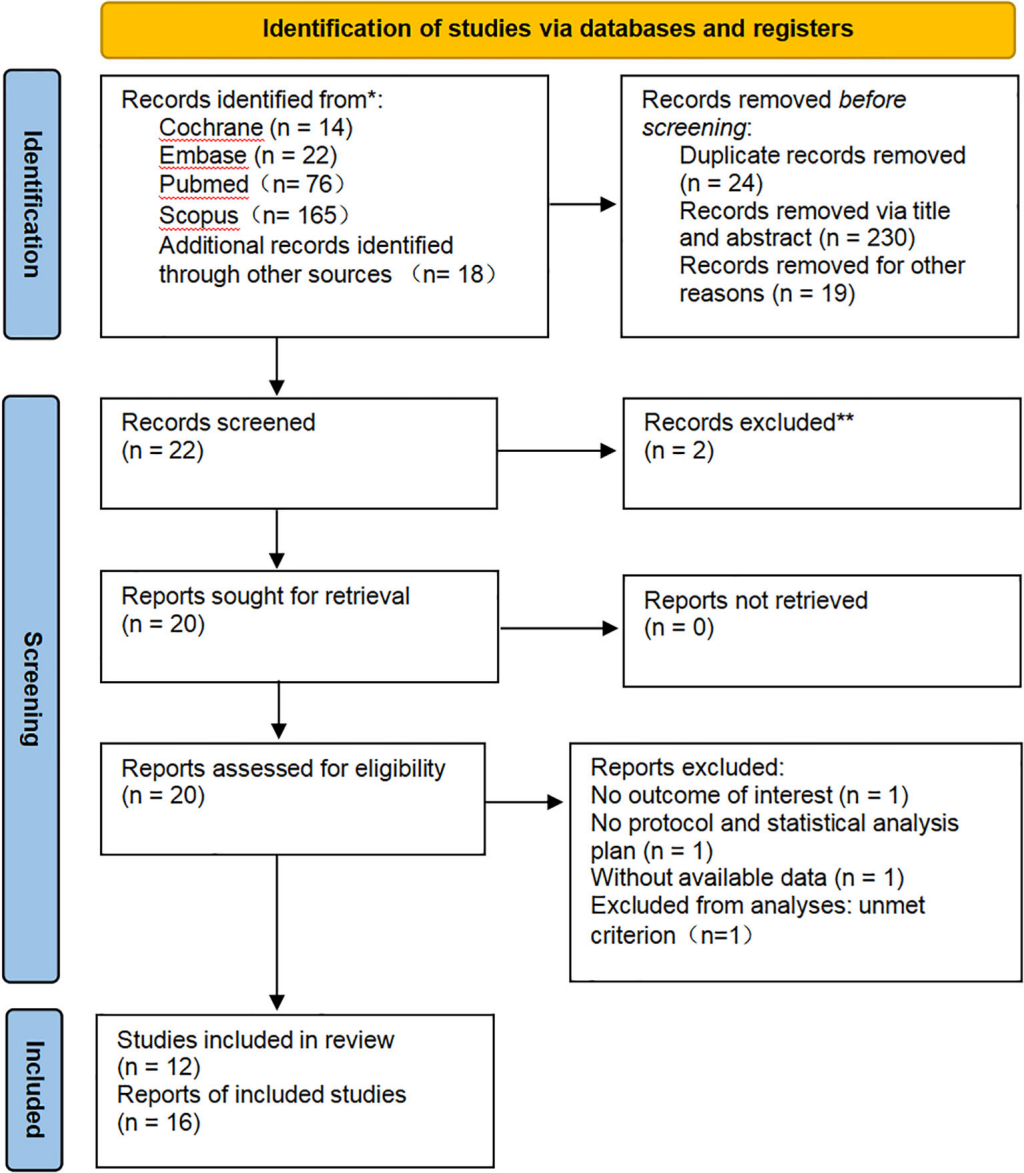
PRISMA diagram: All records were identified through a literature search and citation search. The exclusion process is depicted until the final inclusion of studies.

Based on the predefined search strategy, an initial retrieval yielded 295 records. Following the removal of duplicates, 22 articles underwent eligibility assessment after being screened by title and abstract. Finally, after a full-text review of the remaining articles, 12 studies were included for analysis. All 12 studies were eligible for the efficacy analysis, and 7 of these met the eligibility criteria for the safety analysis. A summary of the specific selection steps is presented in **Figure 1**.

Of the eligible studies, 3 were RCTs and 9 were single-arm studies. The three RCTs of GATSBY, DESTINY-Gastric01, DESTINY-Gastric04 (20, 25, 26) and the three single-arm studies of DS8201-A-U205, DG-06, Zhang et al. (2022) phase Ib dose-expansion trial of ARX788 (21, 27, 28) were specifically designed for HER2-positive advanced gastric cancer (AGC) or GEJ cancers, and the remaining studies included at least one cohort of patients with AGC or GEJ cancers. For all of these trials, enrolled patients were required to receive at least 1 line of standard treatment in an advanced setting. Regarding the number of previously treated lines in advanced or metastatic settings, 92.4% of patients in DS8201-A-U205 received 1 line of antineoplastic therapy (21), which was at least 1 line in the GATSBY, DESTINY-Gastric01, and DESTINY-Gastric04 trials (20, 25, 26), and 55% of patients in Meric-Bernstam et al. (2023) phase I trial of PF-06804103 experienced no less than 6 lines (29). There were significant differences in pretreatment between trials. In addition, in the case of DESTINY-Gastric01, DESTINY-Gastric04 trial, DS8201-A-J101, DS8201-A-U205, Pegram et al. (2021) phase I trial of MEDI4276 (NCT02576548), all included patients received the previous trastuzumab-containing HER2-targeted therapy (20, 21, 26, 30, 31).

Our study evaluated patients with locally advanced or metastatic GC who received ADC monotherapy. The intervention protocol involved ADC drugs including T-DXd, Trastuzumab emtansine (T-DM1), DP303c, RC48, MEDI4276, SYD985, A166, ARX788, PF-06804103. **Table 1** reports the main trial characteristics.

**Table 1:** Design and characteristics of included studies in the meta-analysis.

Clinical trial	Study type	Phase	Sample size	Proportion of HER2+ GC or GEJA, n (%)	Age, year	Previous therapy lines	Intervention	Median follow-up time, months
DS8201-A-J101 (30)^a^	Single-arm	I	274	44 (16%)	68.0 (IQR: 62.5–72.0)	median of 3.0 lines (IQR: 2.0-5.0)	T-DXd	5.5 months (IQR 2.8–13.1)
Zhang et al. (2024) (32)^b^	Single-arm	Ia	94	9 (9.6%)	51.5 (range: 28-73)	≥1 lines: 94(100%)	DP303c	12.0months (range 1.7–35.2)
DS8201-A-U205 (21)^a^	Single-arm	II	79	79 (100%)	60.7 (IQR: 52.0-68.3)	≥1 lines: 79(100%)	T-DXd	10.2 months (IQR 5.6–12.9)
Xu et al. (2021) (33)^b^	Single-arm	I	57	47 (82.5%)	59 (range: 28-75)	1 line: 24(42.1%) 2 lines: 18(31.6%) ≥3 lines: 15(26.3%)	RC48	NA
Pegram et al. (2021) (31)^a^	Single-arm	I	47	15 (31.9%)	66 (range: 44-76)	median of 4 lines (range: 2–8)	MEDI4276	8 months (range 0.7–30.6)^b^
Banerji et al. (2019) (34)^a^	Single-arm	Ib	146	17 (11.6%)	61 (range:52-68)	median of 4.0 lines (IQR: 3-7)	SYD985	5.0 months (IQR 2.9-7.6)^b^
Zhang et al. (2022) (28)^a^	Single-arm	Ib	30	30 (100%)	57 (range: 26-72)	1 lines: 18(60%) ≥2 lines: 12(40%)	ARX788	10 months (95%CI: 6.5-15.9)
Meric-Bernstam et al. (2023) (29)^b^	Single-arm	IA	47	22 (46.8%)	58.0 (range: 32-74)	median of 5.0 lines (range: 1.0–18.0)	PF-06804103	NA
DG-06 (27)^a^	Single-arm	II	95	95 (100%)	65.8% of patients <65	median of 2 lines (range 2-6)	T-DXd	8.0 months (IQR 6.0–13.2)
GATSBY (25)^a^	Randomized	II/III	415	415 (100%)	62.0 (range: 19-79)	1 line: 415(100%)	T-DM1	17.5 months (IQR 12.1–23.0)
DESTINY-Gastric01 (20)^a^	Randomized	II	188	188 (100%)	65 (range: 34-82)	median of 2 lines (range: 2-9)	T-DXd	NA
DESTINY-Gastric04 (26)^a^	Randomized	III	494	494 (100%)	63.2(NA)	≥ 1 line: 494(100%)	T-DXd	16.8 months (95%CI: 14.0-20.0)

GC, gastric cancer; GEJA, gastroesophageal junction adenocarcinoma; T-DXd, trastuzumab deruxtecan; T-DM1, trastuzumab emtansine; RC48, disitamab vedotin; SYD985, trastuzumab duocarmazine; NA, data not available; CI, confidence interval; IQR, interquartile range;

a The study separately reported the baseline characteristics of the patient population with GC or GEJA.

b The data are for the overall study cohort.

### 3.2 Quality assessment

According to the ROBINS-I tool (**Supplementary Table S1**), among the included single group studies, DS8201-A-U205 and Banerji et al. (2019) were assessed as severe, and Pegram et al. (2021) trial was assessed as low risk of bias. The remaining six single group studies were all considered to have moderate risk. According to the RoB 2.0 tool (**Supplementary Table S2**), among the included RCTs, the Meric-Bernstam et al. (2023) trial was assessed as having a high risk of bias, and the remaining three were labeled as Some concerns.

The funnel plot analysis of the ORR showed a suspicious publication bias, which may be related to the tendency of small studies to report higher efficacy values (**Supplementary Figure S2**). Further Harbord regression analysis confirmed the existence of small-study effects, requiring caution in interpreting the results and combining sensitivity analysis to observe the stability of the results (**Supplementary Figure S3**). The results of the Begg test suggested a risk of systemic bias, but did not meet the statistically significant criteria (Kendall’s tau = -0.4242, p = 0.0629).

### 3.3 Efficacy

Of all the included studies, 12 reported data on the effectiveness of ADC interventions, with ORR selected as the primary clinical activity outcome for analysis. Descriptive statistics were used to present ORR results for 3 RCTs meeting the effectiveness criteria; a single-group Meta-analysis was performed regarding the remaining 9 single-arm studies.

A descriptive analysis of ORR data from three RCT studies (GATSBY, DESTINY-Gastric01, DESTINY-Gastric04) is reported in **Table 2**. The overall ORR of the intervention group (37.1%, 207/558) performed better than that of the control group (24.6%, 97/395). Notable variation in efficacy was observed across trials, with the DESTINY-Gastric01 intervention group (51.3%, 61/119) demonstrating the largest improvement in ORR compared with the control group (14.3%, 8/56). A potential correlation between GC ratio and ORR improvement was noted. The most significant ORR advantage was observed in DESTINY-Gastric01 with the highest GC ratio, while the efficacy difference in GATSBY with a lower GC ratio and DESTINY-Gastric04 was relatively convergent (**Supplementary Figures S1A–C**). These findings suggest that the intervention program may have a more prominent clinical benefit for people with specific pathological characteristics (such as high GC ratio).

**Table 2:** Summary of ORR in 3 included randomized controlled trials (N = 1,389).

Clinical trial	Intervention Cohort n/ORR(%)	Control Cohort n/ORR(%)	Relative Risk (95% CI)	GC Prevalence in the Cohort
GATSBY	204/42(20.6%)	102/20(19.6%)	1.05(0.65–1.70)	68.1%
DESTINY-Gastric01	119/61(51.3%)	56/8(14.3%)	3.59(1.82–7.08)	87.2%
DESTINY-Gastric04	235/104(44.3%)	237/69(29.1%)	1.52(1.18–1.96)	61.1%

**Figure 2:**
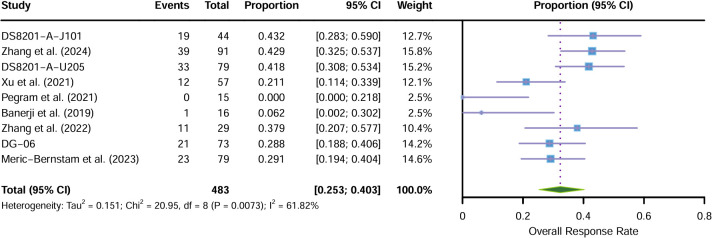
Forest plot: Single-group rate meta-analysis of objective response rate (ORR) in patients with advanced or metastatic HER2-positive gastric cancer treated with ADC.

Nine single-arm trials were pooled using a random-effects model (DerSimonian-Laird method) in which studies with zero events were adjusted for continuity by + 1, as shown in **Figure 2**). The pooled ORR was 32.32% [95% CI: 0.2529; 0.4026]. However, individual study ORRs exhibited a wide range, from 0.0% (Pegram et al. (2021); 95% CI: 0.00-0.22) to 43.2% (DS8201-A-J101 trial; 95% CI: 0.28; 0.59). Since significant heterogeneity was confirmed (I^2^ = 61.82%, p < 0.01), more reliable random effects models have been adopted. Given that results for certain ADCs are derived from single studies with limited sample sizes, these specific estimates should be interpreted with caution. The observed heterogeneity necessitates further exploration of potential sources, such as variations in ADC types and patient baseline characteristics (addressed in subsequent subgroup analyses).

### 3.4 Subgroup analysis

To investigate potential sources of the observed heterogeneity, we performed subgroup analyses based on several key study-level characteristics.

#### 3.4.1 GC patients enrollment proportion

In the nine GC-dedicated cohort studies, the pooled effect size of the ORR was 34.0% (95% CI: 25.1%–44.2%), with substantial heterogeneity observed (I² = 84.1%, Tau² = 0.303, P < 0.0001). In the three studies with cohorts without independent GC data, the pooled ORR was 31.1% (95% CI: 20.1%–44.7%) and was also heterogeneous (I² = 75.1%, Tau² = 0.199, P = 0.0180). The results of the test for subgroup differences showed that there was no statistically significant difference in the response rate between the two groups (Chi² = 0.13, P = 0.7185; **Supplementary Figure S5A**), suggesting that the inclusion of non-GC cohorts was not a primary driver of the overall heterogeneity.

#### 3.4.2 Prior therapeutic lines

All studies provided statistical data on the number of previous antineoplastic treatment lines for the included patients. Among studies with high exposure intensity, the combined ORR effect size was 30.8% (95% CI: 19.5%–44.9%), with considerable intra-group heterogeneity (I² = 69.8%, Tau² = 0.271, P = 0.0102). Conversely, in studies with low exposure intensity, the combined ORR was 34.5% (95% CI: 25.3%–45.1%), with high intra-group heterogeneity (I² = 87.1%, Tau² = 0.297, P < 0.0001). The results of the test for subgroup differences showed that there was no statistically notable difference in the response rate between the subgroups (Chi² = 0.20, P = 0.6559; **Supplementary Figure S5B**), which may be related to intra-group heterogeneity or a sample size imbalance between the two groups. The lack of a significant difference, coupled with high heterogeneity within each subgroup, suggests that while prior treatment is a key clinical variable, its influence on ORR may be complex and confounded by other factors.

#### 3.4.3 Drug of intervention

As shown in the **Supplementary Figure S5C**, this analysis revealed marked differences in efficacy among the various drugs. The ORR pooled effect size was 42.5% (95% CI: 35.8% -49.4%) in the 5 studies where the therapeutic agent was T-DXd, with moderate intra-subgroup heterogeneity (I² = 56.9%, Tau² = 0.055, P = 0.0546). In addition, the efficacy varied substantially for other agents: the ORR was 42.9% (95% CI: 32.5% -53.7%) for the intervention with DP303c, 21.1% (95% CI: 11.4% -33.9%) for the trial of the RC48 drug intervention, 0.0% (95% CI: 0.0% -21.8%) for the trial of the MEDI4276 drug intervention, 6.2% (95% CI: 0.2% -30.2%) for the trial of the SYD985 drug intervention, 37.9% (95% CI: 20.7% -57.7%) for the trial of the ARX788 drug intervention, and 20.6% (95% CI: 15.3% -26.8%) for T-DM1 in GATSBY trial, and the ORR in the trial of PF-06804103 drug intervention was 29.1% (95% CI: 19.4% -40.4%). These divergent outcomes strongly indicate that the choice of intervention regimen is a principal source of the heterogeneity observed in the overall analysis.

### 3.5 Safety

Seven studies reported more comprehensive safety outcome measures. A summary of the main safety findings from the included studies is presented in **Table 3**. In the analysis of 841 patients, the incidence of drug-related AEs of any grade was 96.9% (95% CI: 93.8% -98.5%; **Supplementary Figure S6A**). Except for the DESTINY-Gastric01 trial, which did not report data on the frequency of all AEs grade ≥ 3, 55.1% (95% CI: 44.3% -65.5%) of the remaining 716 patients were observed to have an adverse event of grade ≥ 3 (**Supplementary Figures S6B**). Six studies reported the most common drug-related AEs. The most common AE of any grade was nausea (47.7%, 95% CI: 30.9% -65.0%), followed by anemia (33.3%, 95% CI: 21.6% -47.5%) and decreased appetite (30.7%, 95% CI: 16.7% -49.6%; **Supplementary Figures S7A–C**). Among AEs higher than grade 3, the most common was anemia (21.1%, 95% CI: 13.5% -31.4%), followed by neutropenia (15.1%, 95% CI: 6.6% -31.0%; **Supplementary Figures S7D–F**).

**Table 3:** Summary of main safety findings from included studies on anti-HER2 ADCs in advanced or metastatic GC.

Study	Intervention	Patients (N)	Any grade AE (%)	Grade ≥3 AE (%)	Most common any-grade AEs (≥25%)	Most common grade ≥3 AEs (≥10%)
DS8201-A-J101	T-DXd	44	100 (44/44)	64 (28/44)	Nausea (68%) Decreased Appetite (61%) Anemia (41%)	Anemia (30%) Neutropenia (20%) Decreased Platelets (18%)
DS8201-A-U205	T-DXd	79	100 (79/79)	56 (44/79)	Nausea (67%) Vomiting (44%) Fatigue (42%)	Anemia (14%) Nausea (8%) Neutropenia (8%)
DG-06	T-DXd	95	98 (93/95)	74 (70/95)	NA	NA
DESTINY-Gastric01	T-DXd	125	100 (125/125)	NA	Nausea (63%) Neutropenia (63%) Decreased Appetite (60%)	Neutropenia (51%) Anemia (38%) Decreased WBC (21%)
DESTINY-Gastric04	T-DXd	244	93 (227/244)	50 (122/244)	Fatigue (48%) Neutropenia (48%) Nausea (44%)	Neutropenia (29%) Anemia (14%) Thrombocytopenia (9%)
GATSBY	T-DM1	224	97 (218/224)	60 (134/224)	Anemia (36%) Fatigue (30%) Thrombocytopenia (28%)	Anemia (26%) Thrombocytopenia (11%) Hemorrhage (10%)
Zhang et al. (2022)	ARX788	30	93 (28/30)	13 (4/30)	Dry Eye (63%) Decreased Platelets (37%) Increased AST (33%)	Rare (<5%)
Meta-Analysis Pooled	–	841	96.9 [CI 93.8-98.5]	–	–	–

T-DXd, trastuzumab deruxtecan; T-DM1, trastuzumab emtansine; NA, data not available; CI, confidence interval.

### 3.6 Publication bias and sensitivity analysis

The funnel plot of ORR was asymmetrical. Harbord regression showed a curve deviation (**Supplementary Figures S2, S3**). Begg’s test was marginally significant (Kendall’s tau = -0.4242, p = 0.0629). The three results consistently indicated a mild risk of publication bias, which might be related to the unpublished small-sample studies with negative results. To further evaluate the impact of individual study results on the overall results, a sensitivity analysis was conducted (**Supplementary Figure S4**). The analysis results showed that the combined effect size of ORR changed slightly after excluding each trial, which confirmed the robustness and reliability of this meta-analysis.

## 4 Discussion

This systematic review and meta-analysis evaluated patients with HER2-positive GC treated with ADC drugs. The evaluation involved 12 clinical trials, including RCT trials and single-arm trials. The main results of the pooled analysis were as follows: (1) The RCT study reported 37.1% of the overall ORR of the intervention group (24.6% better than the control group), and the combined ORR response rate of the single-arm study was 32.32% [95% CI: 0.2529; 0.4026], showing a significant efficacy advantage. (2) Analysis based on patient characteristics showed that ADC treatment seemed to benefit patients with HER2-positive tumors. Although there was no considerable statistical difference in ORR levels between the GC-dedicated cohort and the non-GC-dedicated cohort (GC-dedicated cohort: 34.0%, 95% CI: 25.1% -44.2%; non-GC-dedicated cohort: 31.1%, 95% CI: 20.1% -44.7%; P = 0.7185), the three RCT studies still showed that a higher GC proportion was partially correlated with ORR improvement. Its treatment effect was not vitally affected by the number of previous treatment lines. At the same time, there was evident heterogeneity in efficacy between different anti-HER2 ADC drugs. (3) Regarding safety, acceptable AEs were observed in the study, primarily related to the gastrointestinal and hematologic systems. The most frequent AEs of any grade were nausea (47.7%) and anemia (33.3%). Among grade ≥3 AEs, the most common were anemia (21.1%) and neutropenia (15.1%).

HER2 is a critical biomarker and therapeutic target in GC, with overexpression observed in approximately 15-20% of cases. The identification of this target has revolutionized the treatment paradigm for HER2-positive GC, establishing the first successful targeted therapy and laying the foundation for the development of subsequent drugs (35, 36).

The Phase III randomized ToGA trial was the first to demonstrate that anti-HER2 therapy (trastuzumab plus chemotherapy) significantly prolongs survival in patients with HER2-positive AGC (12). The results showed a significant improvement in median overall survival (OS) for the combination therapy group, extending it to 13.8 months from 11.1 months in the chemotherapy-alone group. This trial directly led to the approval of trastuzumab as the world’s first anti-HER2 targeted agent for GC, reshaping the first-line treatment landscape (37).

The GATSBY study was a pivotal Phase II/III clinical trial representing an early exploration of the efficacy of ADCs in HER2-positive GC (38). It was designed to evaluate T-DM1, an ADC with established efficacy in HER2-positive breast cancer, for later-line treatment of HER2-positive GC. However, the trial’s primary finding was disappointing: compared with standard chemotherapy (taxane), T-DM1 failed to demonstrate a significant improvement in either OS (7.9 vs. 8.6 months) or progression-free survival (PFS; 2.7 vs. 2.9 months) (25). The negative results of the GATSBY trial delineated the limitations of T-DM1 in this indication, highlighting issues such as the high degree of tumor heterogeneity and the potential for dynamic changes in HER2 expression status following first-line therapy (39). The setbacks from GATSBY spurred the research and development of a new generation of ADCs, leading to the creation of T-DXd, which incorporates both a “bystander effect” and a “high-potency payload” (40–42).

Subsequently, the DESTINY-Gastric01 trial marked another significant breakthrough (20). This study evaluated patients with HER2-positive GC who had progressed on two or more prior regimens, including trastuzumab. It compared the efficacy of T-DXd against the physician’s choice of chemotherapy (irinotecan or paclitaxel). The results were compelling, demonstrating significantly superior outcomes in the T-DXd arm for both ORR (51% vs. 14%) and median OS (12.5 vs. 8.4 months) compared to the control arm. The remarkable performance of T-DXd in the DESTINY-Gastric01 study, in stark contrast to the results of the GATSBY trial (38), showcased the profound potential of the next-generation ADC platform to overcome resistance to existing HER2-targeted therapies, heralding a new era in the treatment of HER2-positive GC.

However, the clinical benefits of various ADCs in HER2-positive GC appear to be stratified. This phenomenon may reflect a delicate balance between efficacy and toxicity, driven by the distinct molecular architecture of each ADC (42). T-DM1 exhibited a limited ORR of only 24%, a result attributed to its design, specifically a non-cleavable linker and a relatively modest drug-to-antibody ratio (DAR) of approximately 3.5 (25). The non-cleavable linker requires lysosomal degradation of the entire antibody to release its payload (a lysine-linker-DM1 complex), which then has limited membrane permeability. This design significantly curtails the “bystander effect”—the ability to kill adjacent HER2-negative tumor cells—a critical factor in the context of heterogeneous HER2 expression in gastric cancer. In contrast, T-DXd’s high DAR of approximately 8, coupled with a tumor-selective cleavable linker and a highly potent, membrane-permeable DXd payload, achieves precise targeted delivery and a powerful bystander effect. This combination has led to a remarkable 51% ORR in the third-line treatment of HER2-positive GC (20). New-generation ADCs such as ARX788 improve homogeneity through the use of site-specific conjugation technology, which yields a more uniform product with predictable pharmacokinetics, achieving a confirmed ORR of 37.9% (28). MEDI4276, an ADC designed with a dual payload, aimed to further enhance tumor cell internalization and cytolysis, but its high payload toxicity limited the drug dosage, and the final efficacy did not meet expectations (31). On the other hand, these design choices also dictate differences in the safety of ADC therapies. The toxicity profile is strongly correlated with both the payload’s mechanism of action and the linker’s characteristics. T-DXd’s topoisomerase inhibitor, DXd, induces a risk of interstitial lung disease (ILD), while microtubule inhibitor-based payloads ADCs like RC48 and SYD985 are more likely to cause neurotoxicity and myelosuppression (33, 34, 43). Linker stability is also a critical determinant. Cleavable linkers (like T-DXd’s GGFG peptide) selectively release the payload in the tumor microenvironment but risk some premature cleavage in plasma, potentially increasing off-target toxicity. Non-cleavable linkers (as in T-DM1) rely on lysosomal degradation within the target cell for payload release, which may enhance safety by reducing systemic exposure to the free payload but concurrently diminishes the bystander effect in the context of tumor heterogeneity (44).

This meta-analysis confirms the clinical benefit of ADC monotherapy in patients with HER2-positive locally advanced or metastatic GC. Among the evaluated agents, Trastuzumab deruxtecan (T-DXd) has demonstrated excellent and robust efficacy across multiple clinical studies we included (including a pivotal phase I study, DS8201-A-J101, and multiple phase II/III trials, DESTINY-Gastric01, DS8201-A-U205, DESTINY-Gastric04), showing a significant trend of ORR improvement with moderate intra-subgroup heterogeneity. The results of the pivotal phase III clinical trial DESTINY-Gastric04 are particularly outstanding. In this study, the median duration of treatment was 5.4 months, and the median follow-up time was 16.8 months. The median duration of response (DoR) of patients receiving T-DXd reached 7.4 months, and the median PFS was 6.7 months. Based on this strong evidence, T-DXd currently shows the most promise in clinical practice and can be considered a primary or preferred recommendation for this patient population. In addition, Zhang et al. (2022) single-arm study evaluated the intervention effect of the ADC drug ARX788, resulting in an ORR of 37.9% and a PFS of 4.1 months over a 10-month follow-up period. Zhang et al. (2024), a single-arm study evaluating the intervention effect of DP303c, yielded an ORR of 42.9% and a PFS of 4.4 months at a median follow-up of 12 months. Meric-Bernstam et al. (2023) reported an ORR of 29.1% and a PFS of 5.5 months for PF-06804103 intervention; its confidence interval did not overlap with T-DXd (upper limit 40.4% vs. lower limit 35.8%), suggesting efficacy-level differences. These three ADCs can be considered as promising second-line or later-line treatment options, although their definitive clinical positioning requires further validation through larger, phase III randomized controlled trials. In contrast, T-DM1 and RC48 showed limited ORR (20.6% and 21.1%) in the included trials and reported PFS of 2.7 and 3.5 months, respectively. Therefore, while they may still have utility in specific scenarios, their ranking in a potential hierarchy of ADC recommendations would likely be lower than T-DXd and other emerging ADCs. The sample sizes of MEDI4276 and SYD985 subgroups were too small and the response rate was extremely low, and their effect sizes were only exploratory. It is noteworthy that the inclusion of cohorts not specific to gastric cancer may have contributed to the substantial heterogeneity across these studies. Furthermore, the absence of reconfirmed HER2 molecular marker expression following prior treatments could confound the statistical analysis of patient outcomes under ADC intervention.

At present, the treatment of HER2-positive GC faces some important unresolved problems, including treatment resistance caused by tumor heterogeneity (37), insufficient response rate to targeted therapy (25, 45), lack of back-line treatment strategies, and unclear benefits of immune checkpoint inhibitors for this patient population (46). These problems are related to the unique biological characteristics of HER2-positive GC. The development of new ADC drugs is expected to break through the following bottlenecks: killing adjacent HER2-negative cells with the bystander effect to overcome spatial heterogeneity (47); improving HER2 low expression resistance (37); reversing secondary resistance and improving the efficacy of later-line therapy. For new ADCs that have initially proved the robustness of efficacy in previous trials, it is necessary to further verify the efficacy advantages and safety of drugs in specific treatment lines, compared with standard chemotherapy.

Regrettably, current research on ADCs for the treatment of HER2-positive GC is confronted by several persistent challenges. We lack biomarkers that can accurately predict the efficacy of ADCs, and the existing HER2 classification by IHC/FISH is insufficient for this purpose. At the same time, the off-target toxicity of ADC may restrict the improvement of efficacy, resulting in a narrow therapeutic window (48). Some combination regimens of ADC with chemotherapy or immunotherapy show excellent therapeutic potential, but their synergy effect still needs further evidence-based support.

In terms of safety, the included trials reported a high pooled incidence of any-grade AEs, which were predominantly gastrointestinal in nature. Regarding hematological safety, anemia remained the most common hematologic toxicity-related clinical burden in ADC therapy, with an incidence of 33.3%, highlighting the need to strengthen management strategies for anemia-related events. The pooled incidence of grade ≥3 AEs reached 55.1%. The high incidence of anemia (21.1%) may reflect significant treatment-related toxicity, impacting patient tolerability. Fortunately, the occurrence rates of neutropenia (15.1%) and leukopenia (8.1%) were lower than those reported for conventional chemotherapy (12, 49), suggesting that some AEs are manageable and that a degree of safety optimization in terms of myelosuppression can be achieved. It is important to note that the presence of zero-event studies in the statistical data may introduce bias into the pooled results.

This study has several limitations that warrant consideration. Firstly, the analysis utilized the ORR as the primary endpoint. The potential for publication bias concerning ORR outcomes was indicated by funnel plot asymmetry, which was further corroborated by both Begg’s test and Harbord’s regression analysis. This suggests a possible overestimation of the pooled treatment effect due to an underreporting of small-sample studies with negative results. Secondly, a major limitation of this meta-analysis is the predominance of single-arm, non-randomized studies (nine of the included trials). The inherent absence of a concurrent control group in such designs makes it challenging to distinguish the therapeutic effects of the ADCs from the natural disease progression or the impact of confounding variables. This design is highly susceptible to selection bias, and the lack of blinding may introduce performance and detection biases. These issues are compounded by the small sample sizes characteristic of these early-phase trials, which not only limit the statistical power of individual studies but also increase the risk that our pooled estimates are disproportionately influenced by single-study results. While we employed a random-effects model to account for anticipated heterogeneity, and our sensitivity analysis confirmed the robustness of the pooled ORR, these statistical methods cannot fully compensate for the inherent biases of the primary study designs. Encouragingly, the sensitivity analysis demonstrated that the ORR effect size was robust against the exclusion of any single study. Regarding the management of heterogeneity, substantial unexplained variability remained even after subgroup analyses based on the proportion of enrolled GC patients, the number of prior treatment lines, and the specific investigational drug. Furthermore, with the exception of the T-DXd subgroup, the remaining drug subgroups consisted of only a single study, which severely constrains the statistical power for meaningful indirect cross-drug comparisons.

Based on the limitations identified in this meta-analysis, future research on ADCs in HER2-positive advanced GC should prioritize adequately powered RCTs. These trials are essential, first to definitively establish the efficacy of novel ADCs against the standard of care, and subsequently, to conduct head-to-head comparisons to determine optimal agent selection and sequencing. Crucially, such studies must employ robust primary endpoints like progression-free and overall survival, moving beyond a reliance on response rates. Furthermore, to elucidate the substantial clinical heterogeneity observed, comprehensive translational and biomarker analyses should be integral to these trials. Investigating predictive markers, including levels of HER2 expression and co-occurring molecular alterations, is imperative for patient stratification and the development of personalized therapeutic strategies. Adopting innovative designs, such as international platform trials, could accelerate these efforts by enabling the simultaneous evaluation of multiple agents and biomarkers.

## 5 Conclusions

In general, our systematic review and meta-analysis support that ADC may be a promising treatment option for HER2-positive locally advanced or metastatic GC. The combined ORR effect value of all included trials reached 33.4%, which is better than the performance of traditional chemotherapy in the treatment of HER2-positive GC. Different types of ADC drugs show obvious stratification in therapeutic effects, but their therapeutic effects may be less restricted by the number of previous treatment lines. However, studies have also observed digestive system and hematological system-related toxicity caused by ADC drugs, suggesting that monitoring and management of AEs in clinical application are crucial. At present, more studies are underway to further clarify the antitumor activity of new ADC drugs and explore their efficacy in combination with other therapies. These studies will provide higher-level evidence-based medical evidence for optimizing treatment strategies for patients with HER2-positive GC.

## Data Availability

The original contributions presented in the study are included in the article/**Supplementary Material**. Further inquiries can be directed to the corresponding author.

## References

[1] Yang WJ Zhao HP Yu Y Wang JH Guo L Liu JY . Updates on global epidemiology, risk and prognostic factors of gastric cancer. World J Gastroenterol. (2023) 29:2452–68. doi: 10.3748/wjg.v29.i16.2452, PMID: 37179585 PMC10167900

[2] Sundar R Nakayama I Markar SR Shitara K van Laarhoven HWM Janjigian YY . Gastric cancer. Lancet (lond Engl). (2025) 405:2087–102. doi: 10.1016/S0140-6736(25)00052-2, PMID: 40319897

[3] Guan WL He Y Xu RH . Gastric cancer treatment: recent progress and future perspectives. J Hematol Oncol. (2023) 16:57. doi: 10.1186/s13045-023-01451-3, PMID: 37245017 PMC10225110

[4] Liu J Zhu T Zhao R Ren W Zhao F Liu J . Elucidating molecular mechanisms and therapeutic synergy: irreversible HER2-TKI plus T-Dxd for enhanced anti-HER2 treatment of gastric cancer. Gastric Cancer. (2024) 27:495–505. doi: 10.1007/s10120-024-01478-6, PMID: 38386239 PMC11016512

[5] Cai J Wang W Cong D Bai Y Zhang W . Development of treatment strategies for advanced HER2-positive gastric cancer: Insights from clinical trials. Crit Rev Oncol Hematol. (2025) 207:104617. doi: 10.1016/j.critrevonc.2025.104617, PMID: 39805409

[6] Torre LA Siegel RL Ward EM Jemal A . Global cancer incidence and mortality rates and trends–an update. Cancer epidemiology Biomarkers Prev. (2016) 25:16–27. doi: 10.1158/1055-9965.EPI-15-0578, PMID: 26667886

[7] Abrahao-MaChado LF Scapulatempo-Neto C . HER2 testing in gastric cancer: An update. WJG. (2016) 22:4619. doi: 10.3748/wjg.v22.i19.4619, PMID: 27217694 PMC4870069

[8] Zhao D Klempner SJ Chao J . Progress and challenges in HER2-positive gastroesophageal adenocarcinoma. J Hematol Oncol. (2019) 12:50. doi: 10.1186/s13045-019-0737-2, PMID: 31101074 PMC6525398

[9] Shao Q Deng J Wu H Huang Z . HER2-positive gastric cancer: from targeted therapy to CAR-T cell therapy. Front Immunol. (2025) 16:1560280. doi: 10.3389/fimmu.2025.1560280, PMID: 40181988 PMC11966040

[10] Kawakami H Nakanishi K Makiyama A Konishi H Morita S Narita Y . Real-world effectiveness and safety of trastuzumab-deruxtecan in Japanese patients with HER2-positive advanced gastric cancer (EN-DEAVOR study). Gastric Cancer. (2025) 28:51–61. doi: 10.1007/s10120-024-01555-w, PMID: 39387986 PMC11706843

[11] Cammarota A Woodford R Smyth EC . Targeting HER2 in gastroesophageal cancer: A new appetite for an old plight. Drugs. (2025) 85:361–83. doi: 10.1007/s40265-024-02132-2, PMID: 39843758

[12] Bang YJ Van Cutsem E Feyereislova A Chung HC Shen L Sawaki A . Trastuzumab in combination with chemotherapy versus chemotherapy alone for treatment of HER2-positive advanced gastric or gastro-oesophageal junction cancer (ToGA): a phase 3, open-label, randomised controlled trial. Lancet. (2010) 376:687–97. doi: 10.1016/S0140-6736(10)61121-X, PMID: 20728210

[13] Shitara K Bang YJ Iwasa S Sugimoto N Ryu MH Sakai D . Trastuzumab deruxtecan in HER2-positive advanced gastric cancer: exploratory biomarker analysis of the randomized, phase 2 DESTINY-Gastric01 trial. Nat Med. (2024) 30:1933–42. doi: 10.1038/s41591-024-02992-x, PMID: 38745009 PMC11271396

[14] Yoon J Oh DY . HER2-targeted therapies beyond breast cancer - an update. Nat Rev Clin Oncol. (2024) 21:675–700. doi: 10.1038/s41571-024-00924-9, PMID: 39039196

[15] Yoshihara K Kobayashi Y Endo S Fukae M Hennig S Kastrissios H . Trastuzumab deruxtecan dosing in human epidermal growth factor receptor 2-positive gastric cancer: population pharmacokinetic modeling and exposure–response analysis. J Clin Pharmacol. (2023) 63:1232–43. doi: 10.1002/jcph.2295, PMID: 37393579

[16] DiPeri TP Evans KW Raso MG Zhao M Rizvi YQ Zheng X . Adavosertib enhances antitumor activity of trastuzumab deruxtecan in HER2-expressing cancers. Clin Cancer Res. (2023) 29:4385–98. doi: 10.1158/1078-0432.CCR-23-0103, PMID: 37279095 PMC10618648

[17] Wang J Liu Y Zhang Q Li W Feng J Wang X . Disitamab vedotin, a HER2-directed antibody-drug conjugate, in patients with HER2-overexpression and HER2-low advanced breast cancer: a phase I/Ib study. Cancer Commun. (2024) 44:833–51. doi: 10.1002/cac2.12577, PMID: 38940019 PMC11260767

[18] Li BT Michelini F Misale S Cocco E Baldino L Cai Y . HER2-mediated internalization of cytotoxic agents in ERBB2 amplified or mutant lung cancers. Cancer Discov. (2020) 10:674–87. doi: 10.1158/2159-8290.CD-20-0215, PMID: 32213539 PMC7196485

[19] Wei Q Yang T Zhu J Zhang Z Yang L Zhang Y . Spatiotemporal quantification of HER2-targeting antibody-drug conjugate bystander activity and enhancement of solid tumor penetration. Clin Cancer Res. (2024) 30:984–97. doi: 10.1158/1078-0432.CCR-23-1725, PMID: 38113039

[20] Shitara K Bang YJ Iwasa S Sugimoto N Ryu MH Sakai D . Trastuzumab deruxtecan in previously treated HER2-positive gastric cancer. N Engl J Med. (2020) 382:2419–30. doi: 10.1056/NEJMoa2004413, PMID: 32469182

[21] Van Cutsem E di Bartolomeo M Smyth E Chau I Park H Siena S . Trastuzumab deruxtecan in patients in the USA and Europe with HER2-positive advanced gastric or gastroesophageal junction cancer with disease progression on or after a trastuzumab-containing regimen (DESTINY-Gastric02): primary and updated analyses from a single-arm, phase 2 study. Lancet Oncol. (2023) 24:744–56. doi: 10.1016/S1470-2045(23)00215-2, PMID: 37329891 PMC11298287

[22] Shah MA Kennedy EB Alarcon-Rozas AE Alcindor T Bartley AN Malowany AB . Immunotherapy and targeted therapy for advanced gastroesophageal cancer: ASCO guideline. J Clin Oncol. (2023) 41:1470–91. doi: 10.1200/JCO.22.02331, PMID: 36603169

[23] Samantasinghar A Sunildutt NP Ahmed F Soomro AM Salih ARC Parihar P . A comprehensive review of key factors affecting the efficacy of antibody drug conjugate. BioMed Pharmacother. (2023) 161:114408. doi: 10.1016/j.biopha.2023.114408, PMID: 36841027

[24] Nguyen TD Bordeau BM Balthasar JP . Mechanisms of ADC toxicity and strategies to increase ADC tolerability. Cancers (Basel). (2023) 15. doi: 10.3390/cancers15030713, PMID: 36765668 PMC9913659

[25] Thuss-Patience PC Shah MA Ohtsu A Van Cutsem E Ajani JA Castro H . Trastuzumab emtansine versus taxane use for previously treated HER2-positive locally advanced or metastatic gastric or gastro-oesophageal junction adenocarcinoma (GATSBY): an international randomised, open-label, adaptive, phase 2/3 study. Lancet Oncol. (2017) 18:640–53. doi: 10.1016/S1470-2045(17)30111-0, PMID: 28343975

[26] Shitara K Cutsem EV Gümüş M Lonardi S de la Fouchardière C Coutzac C . Trastuzumab deruxtecan or ramucirumab plus paclitaxel in gastric cancer. New Engl J Med. (2025) 393:336–48. doi: 10.1056/NEJMoa2503119, PMID: 40454632

[27] Shen L Chen P Lu J Wan Y Zheng Y Ye F . 172P Trastuzumab deruxtecan (T-DXd) in Chinese patients (pts) with previously treated HER2-positive locally advanced/metastatic gastric cancer (GC) or gastroesophageal junction adenocarcinoma (GEJA): Primary efficacy and safety from the phase II single-arm DESTINY-Gastric06 (DG06) trial. Ann Oncol. (2023) 34:S1542–3. doi: 10.1016/j.annonc.2023.10.307

[28] Zhang Y Qiu MZ Wang JF Zhang YQ Shen A Yuan XL . Phase 1 multicenter, dose-expansion study of ARX788 as monotherapy in HER2-positive advanced gastric and gastroesophageal junction adenocarcinoma. Cell Rep Med. (2022) 3:100814. doi: 10.1016/j.xcrm.2022.100814, PMID: 36384091 PMC9729820

[29] Meric-Bernstam F Calvo E Lee KS Moreno V Park YH Rha SY . Safety and tolerability of a novel anti-HER2 antibody-drug conjugate (PF-06804103) in patients with HER2-expressing solid tumors: A phase 1 dose-escalation study. Mol Cancer Ther. (2023) 22:1191–203. doi: 10.1158/1535-7163.MCT-23-0101, PMID: 37420274 PMC10543980

[30] Shitara K Iwata H Takahashi S Tamura K Park H Modi S . Trastuzumab deruxtecan (DS-8201a) in patients with advanced HER2-positive gastric cancer: a dose-expansion, phase 1 study. Lancet Oncol. (2019) 20:827–36. doi: 10.1016/S1470-2045(19)30088-9, PMID: 31047804

[31] Pegram MD Hamilton EP Tan AR Storniolo AM Balic K Rosenbaum AI . First-in-human, phase 1 dose-escalation study of biparatopic anti-HER2 antibody-drug conjugate MEDI4276 in patients with HER2-positive advanced breast or gastric cancer. Mol Cancer Ther. (2021) 20:1442–53. doi: 10.1158/1535-7163.MCT-20-0014, PMID: 34045233 PMC9398097

[32] Zhang J Du Y Meng Y Liu X Mu Y Liu Y . First-in-human study of DP303c, a HER2-targeted antibody-drug conjugate in patients with HER2 positive solid tumors. NPJ Precis Oncol. (2024) 8:200. doi: 10.1038/s41698-024-00687-7, PMID: 39266619 PMC11393109

[33] Xu Y . Phase I study of the recombinant humanized anti-HER2 monoclonal antibody–MMAE conjugate RC48-ADC in patients with HER2-positive advanced solid tumors. Gastric Cancer. (2021) 24:913–25. doi: 10.1007/s10120-021-01168-7, PMID: 33945049 PMC8205919

[34] Banerji U van Herpen CML Saura C Thistlethwaite F Lord S Moreno V . Trastuzumab duocarmazine in locally advanced and metastatic solid tumours and HER2-expressing breast cancer: a phase 1 dose-escalation and dose-expansion study. Lancet Oncol. (2019) 20:1124–35. doi: 10.1016/S1470-2045(19)30328-6, PMID: 31257177

[35] Kudo T . Advances in the treatment of human epidermal growth factor receptor 2-positive gastric cancer. Int J Clin Oncol. (2024) 29:1220–7. doi: 10.1007/s10147-024-02587-z, PMID: 39083154

[36] Grieb BC Agarwal R . HER2-directed therapy in advanced gastric and gastroesophageal adenocarcinoma: triumphs and troubles. Curr Treat Options Oncol. (2021) 22:88. doi: 10.1007/s11864-021-00884-7, PMID: 34424404 PMC8436174

[37] Zhu Y Zhu X Wei X Tang C Zhang W . HER2-targeted therapies in gastric cancer. Biochim Biophys Acta Rev Cancer. (2021) 1876:188549. doi: 10.1016/j.bbcan.2021.188549, PMID: 33894300

[38] Koganemaru S Shitara K . Antibody-drug conjugates to treat gastric cancer. Expert Opin Biol Ther. (2021) 21:923–30. doi: 10.1080/14712598.2020.1802423, PMID: 32713216

[39] Aoki Y Nakayama I Shitara K . Human epidermal growth factor receptor 2 positive advanced gastric or esophagogastric adenocarcinoma: reflecting on the past to gain a new insights. Curr Oncol Rep. (2025) 27:15–29. doi: 10.1007/s11912-024-01626-2 39753814

[40] Jang JY Kim D Lee NK Im E Kim ND . Antibody-drug conjugates powered by deruxtecan: innovations and challenges in oncology. Int J Mol Sci. (2025) 26:6523. doi: 10.3390/ijms26136523, PMID: 40650299 PMC12250044

[41] Shimozaki K Fukuoka S Ooki A Yamaguchi K . HER2-low gastric cancer: is the subgroup targetable? ESMO Open. (2024) 9:103679. doi: 10.1016/j.esmoop.2024.103679, PMID: 39178538 PMC11386020

[42] Tarantino P Carmagnani Pestana R Corti C Modi S Bardia A Tolaney SM . Antibody-drug conjugates: Smart chemotherapy delivery across tumor histologies. CA Cancer J Clin. (2022) 72:165–82. doi: 10.3322/caac.21705, PMID: 34767258

[43] Wang P Xia L . RC48-ADC treatment for patients with HER2-expressing locally advanced or metastatic solid tumors: a real-world study. BMC Cancer. (2023) 23:1083. doi: 10.1186/s12885-023-11593-9, PMID: 37946161 PMC10636982

[44] Nakada T Sugihara K Jikoh T Abe Y Agatsuma T . The latest research and development into the antibody-drug conjugate, [fam-] trastuzumab deruxtecan (DS-8201a), for HER2 cancer therapy. Chem Pharm Bull (Tokyo). (2019) 67:173–85. doi: 10.1248/cpb.c18-00744, PMID: 30827997

[45] Tabernero J Hoff PM Shen L Ohtsu A Shah MA Cheng K . Pertuzumab plus trastuzumab and chemotherapy for HER2-positive metastatic gastric or gastro-oesophageal junction cancer (JACOB): final analysis of a double-blind, randomised, placebo-controlled phase 3 study. Lancet Oncol. (2018) 19:1372–84. doi: 10.1016/S1470-2045(18)30481-9, PMID: 30217672

[46] Janjigian YY Maron SB Chatila WK Millang B Chavan SS Alterman C . First-line pembrolizumab and trastuzumab in HER2-positive oesophageal, gastric, or gastro-oesophageal junction cancer: an open-label, single-arm, phase 2 trial. Lancet Oncol. (2020) 21:821–31. doi: 10.1016/S1470-2045(20)30169-8, PMID: 32437664 PMC8229851

[47] Jubashi A Nakayama I Koganemaru S Sakamoto N Oda S Matsubara Y . Prognostic and predictive factors for the efficacy and safety of trastuzumab deruxtecan in HER2-positive gastric or gastroesophageal junction cancer. Gastric Cancer. (2025) 28:63–73. doi: 10.1007/s10120-024-01560-z, PMID: 39487862 PMC11706866

[48] Shitara K Baba E Fujitani K Oki E Fujii S Yamaguchi K . Discovery and development of trastuzumab deruxtecan and safety management for patients with HER2-positive gastric cancer. Gastric Cancer. (2021) 24:780–9. doi: 10.1007/s10120-021-01196-3, PMID: 33997928 PMC8205906

[49] Wilke H Muro K Van Cutsem E Oh SC Bodoky G Shimada Y . Ramucirumab plus paclitaxel versus placebo plus paclitaxel in patients with previously treated advanced gastric or gastro-oesophageal junction adenocarcinoma (RAINBOW): a double-blind, randomised phase 3 trial. Lancet Oncol. (2014) 15:1224–35. doi: 10.1016/S1470-2045(14)70420-6, PMID: 25240821

