# Supplementary material for: Antibody-drug conjugates in HER2-positive advanced or metastatic gastric cancer: a systematic review and meta-analysis
Source: Front Oncol. 2025 Oct 29;15:1684873. doi: 10.3389/fonc.2025.1684873 (PMC12605428; doi:10.3389/fonc.2025.1684873)
Supplement: Supplementary file 1 [file SupplementaryFile1.docx]

**Supplementary Material**

**Efficacy and safety of Antibody-drug conjugates in HER2-positive**

**advanced or metastatic gastric cancer: a systematic review and meta-analysis**

Jiayang Li^^[[1]](#footnote-1)^,+^ , Shuangyu Chen^1,+^, Yinying Chai^1,+^, Shengliang Qiu^^[[2]](#footnote-2)^^

Supplementary material – Method

## Supplementary Method 1. Search strategy for PubMed

("Stomach Neoplasms"[MeSH Terms] OR ((stomach[tiab] OR gastric[tiab]) AND (neoplas*[tiab] OR cancer*[tiab] OR carcino*[tiab] OR adenocarcino*[tiab] OR tumor[tiab] OR tumors[tiab] OR tumour[tiab] OR tumours[tiab] OR malig*[tiab])))

AND

("Neoplasm Metastasis"[MeSH Terms] OR "Neoplasm Metastases"[tiab] OR (advanced[tiab] OR stage III[tiab] OR stage IV[tiab] OR stage four[tiab] OR unresectable[tiab] OR inoperable[tiab] ) OR ((metastases[tiab] OR metastasis[tiab] OR metasta*[tiab] OR advanced[tiab]) AND (neoplas*[tiab] OR cancer*[tiab] OR carcino*[tiab] OR adenocarcino*[tiab] OR tumor[tiab] OR tumors[tiab] OR tumour[tiab] OR tumours[tiab] OR malig*[tiab])))

AND

(("Genes, erbB-2"[Mesh] OR "Receptor, ErbB-2"[Mesh]) OR ("ERBB2 protein, human" [Supplementary Concept]) OR ((v-erb-b2 erythroblastic leukemia viral oncogene homolog 2 protein, human[tiab]) OR (HER2 protein, human[tiab]) OR (Neu protein, human[tiab]) OR (EGFR2 protein, human[tiab]) OR (HER-2 protein, human[tiab]) OR (HER2delta16 protein, human[tiab]) OR (epidermal growth factor receptor 2, human[tiab]) OR (human epidermal growth factor receptor 2[tiab]) OR (HER-2[tiab]) OR (HER2[tiab]) OR (ERBB2[tiab])))

AND

(("antibody-drug conjugates"[Title/Abstract] OR "antibody-drug conjugate"[Title/Abstract] OR "ADC"[Title/Abstract] OR "ADCs"[Title/Abstract]))

AND

((Brentuximab Vedotin [Title/Abstract]) OR (Adcetris [Title/Abstract]) OR (SGN-35 [Title/Abstract]) OR (Belantamab Mafodotin [Title/Abstract]) OR (Blenrep [Title/Abstract]) OR (GSK2857916 [Title/Abstract]) OR (Cetuximab Saratolacan [Title/Abstract]) OR (Akalux [Title/Abstract]) OR (Enfortumab Vedotin [Title/Abstract]) OR (Padcev [Title/Abstract]) OR (ASG-22ME [Title/Abstract]) OR (Gemtuzumab Ozogamicin [Title/Abstract]) OR (Mylotarg [Title/Abstract]) OR (CMA-676 [Title/Abstract]) OR (Elahere [Title/Abstract]) OR (Inotuzumab Ozogamicin [Title/Abstract]) OR (Besponsa [Title/Abstract]) OR (CMC-544 [Title/Abstract]) OR (Moxetumomab Pasudotox [Title/Abstract]) OR (Lumoxiti [Title/Abstract]) OR (Polatuzumab Vedotin [Title/Abstract]) OR (Polivy [Title/Abstract]) OR (Sacituzumab Govitecan [Title/Abstract]) OR (Trodelvy [Title/Abstract]) OR (IMMU-132 [Title/Abstract]) OR (Trastuzumab Deruxtecan [Title/Abstract]) OR (Enhertu [Title/Abstract]) OR (DS-8201 [Title/Abstract]) OR (Trastuzumab Emtansine [Title/Abstract]) OR (Kadcyla [Title/Abstract]) OR (TDM-1 [Title/Abstract]) OR (1959-sss-DM3 [Title/Abstract]) OR (A166 [Title/Abstract]) OR (ABBV-085 [Title/Abstract]) OR (ABBV-321 [Title/Abstract]) OR (ABBV-399 [Title/Abstract]) OR (ADCT-301 [Title/Abstract]) OR (ADCT-402 [Title/Abstract]) OR (A-dmDT390-bisFv [Title/Abstract]) OR (Anetumab ravtansine [Title/Abstract]) OR (ARX788 [Title/Abstract]) OR (ATOR-1015 [Title/Abstract]) OR (B003 [Title/Abstract]) OR (BAT8001 [Title/Abstract]) OR (BAY 94-9343 [Title/Abstract]) OR (BAY-943 [Title/Abstract]) OR (BMS-986148 [Title/Abstract]) OR (BT-062 [Title/Abstract]) OR (BT1718 [Title/Abstract]) OR (BT5528 [Title/Abstract]) OR (Camidanlumab tesirine [Title/Abstract]) OR (cofetuzumab pelidotin [Title/Abstract]) OR (CX-2009 [Title/Abstract]) OR (CX-2029 [Title/Abstract]) OR (Datopotamab deruxtecan [Title/Abstract]) OR (depatuxizumab mafodotin [Title/Abstract]) OR (disitamab vedotin [Title/Abstract]) OR (Dolaflexin [Title/Abstract]) OR (DS-1062a [Title/Abstract]) OR (DS-6157a [Title/Abstract]) OR (Enapotamab vedotin [Title/Abstract]) OR (F0002-ADC [Title/Abstract]) OR (HuMax-TF-ADC [Title/Abstract]) OR (IMGN-853 [Title/Abstract]) OR (Indatuximab ravtansine [Title/Abstract]) OR (Ladiratuzumab vedotin [Title/Abstract]) OR (L-DOS47 [Title/Abstract]) OR (Loncastuximab tesirine [Title/Abstract]) OR (Lorvotuzumab Mertansine [Title/Abstract]) OR (MEDI2228 [Title/Abstract]) OR (MEN1309 [Title/Abstract]) OR (MGC018 [Title/Abstract]) OR (Mirvetuximab soravtansine [Title/Abstract]) OR (Mitazalimab [Title/Abstract]) OR (MORAb-202 [Title/Abstract]) OR (Naratuximab emtansine [Title/Abstract]) OR (OBI-999 [Title/Abstract]) OR (Oportuzumab Monatox [Title/Abstract]) OR (patritumab deruxtecan [Title/Abstract]) OR (PF-06647020 [Title/Abstract]) OR (PF-06804103 [Title/Abstract]) OR (PSMA-ADC [Title/Abstract]) OR (RC48-ADC [Title/Abstract]) OR (SAR408701 [Title/Abstract]) OR (Serclutamab talirine [Title/Abstract]) OR (SGN-LIV1A [Title/Abstract]) OR (SHR-A1403 [Title/Abstract]) OR (STRO-001 [Title/Abstract]) OR (SYD985 [Title/Abstract]) OR (TAK-164 [Title/Abstract]) OR (telisotuzumab vedotin [Title/Abstract]) OR (Tisotumab vedotin [Title/Abstract]) OR (Tivdak [Title/Abstract]) OR (Trastuzumab duocarmazine [Title/Abstract]) OR (U3-1402 [Title/Abstract]) OR (XMT-1536 [Title/Abstract]) OR (XMT-1592 [Title/Abstract]))

## Supplementary Method 2. Search strategy for Embase

1. Receptor, ErbB-2/
2. Genes, erbB-2/
3. (human epidermal growth factor*).ti,ab,kf.
4. (HER2 or "HER2+" or HER-2 or "HER-2+" or "HER/neu" or "HER2/neu").ti,ab,kf.
5. (ERBB2 or ERBB-2 or erb-b2 or "erbb2/neu" or "erbb-2/neu" or "erb-b2/neu").ti,ab,kf.
6. or/1-5
7. Stomach Tumor/ or exp Stomach Neoplasms/
8. exp Stomach/
9. (stomach* or gastric).ti,ab,kf.
10. 8 or 9
11. exp Neoplasms/
12. (neoplas* or cancer* or carcino* or adenocarcino* or carcinoma or malig* or tumor* or tumour*).ti,ab,kf.
13. 11 or 12
14. 10 and 13
15. 7 or 14
16. exp Metastasis/ or exp Neoplasm Metastasis/ or Metastasis/ or Neoplasm Metastasis/
17. ((neoplas* or cancer* or carcino* or adenocarcino* or carcinoma or malig* or tumor* or tumour*) and (metastases or metastasis)).ti,ab,kf.
18. or/16-17
19. 15 and 18
20. (exp Antibody drug/) or (antibody drug*)
21. (exp Conjugate'/) or (conjugate*)
22. 20 and 21
23. (exp Antibody/) or (antibody*)
24. (exp Drug/) or (drug*)
25. 23 and 24 and 21
26. 22 or 25
27. (((antibody drug* or (antibody* and drug*)) and conjugate*) or ADC* or (antibody conjugate*)).ti,ab,kf.
28. ((Brentuximab Vedotin) OR (Adcetris) OR (SGN-35) OR (Belantamab Mafodotin) OR (Blenrep) OR (GSK2857916) OR (Cetuximab Saratolacan) OR (Akalux) OR (Enfortumab Vedotin) OR (Padcev) OR (ASG-22ME) OR (Gemtuzumab Ozogamicin) OR (Mylotarg) OR (CMA-676) OR (Elahere) OR (Inotuzumab Ozogamicin) OR (Besponsa) OR (CMC-544) OR (Moxetumomab Pasudotox) OR (Lumoxiti) OR (Polatuzumab Vedotin) OR (Polivy) OR (Sacituzumab Govitecan) OR (Trodelvy) OR (IMMU-132) OR (Trastuzumab Deruxtecan) OR (Enhertu) OR (DS-8201) OR (Trastuzumab Emtansine) OR (Kadcyla) OR (TDM-1) OR (1959-sss-DM3) OR (A166) OR (ABBV-085) OR (ABBV-321) OR (ABBV-399) OR (ADCT-301) OR (ADCT-402) OR (A-dmDT390-bisFv) OR (Anetumab ravtansine) OR (ARX788) OR (ATOR-1015) OR (B003) OR (BAT8001) OR (BAY 94-9343) OR (BAY-943) OR (BMS-986148) OR (BT-062) OR (BT1718) OR (BT5528) OR (Camidanlumab tesirine) OR (cofetuzumab pelidotin) OR (CX-2009) OR (CX-2029) OR (Datopotamab deruxtecan) OR (depatuxizumab mafodotin) OR (disitamab vedotin) OR (Dolaflexin) OR (DS-1062a) OR (DS-6157a) OR (Enapotamab vedotin) OR (F0002-ADC) OR (HuMax-TF-ADC) OR (IMGN-853) OR (Indatuximab ravtansine) OR (Ladiratuzumab vedotin) OR (L-DOS47) OR (Loncastuximab tesirine) OR (Lorvotuzumab Mertansine) OR (MEDI2228) OR (MEN1309) OR (MGC018) OR (Mirvetuximab soravtansine) OR (Mitazalimab) OR (MORAb-202) OR (Naratuximab emtansine) OR (OBI-999) OR (Oportuzumab Monatox) OR (patritumab deruxtecan) OR (PF-06647020) OR (PF-06804103) OR (PSMA-ADC) OR (RC48-ADC) OR (SAR408701) OR (Serclutamab talirine) OR (SGN-LIV1A) OR (SHR-A1403) OR (STRO-001) OR (SYD985) OR (TAK-164) OR (telisotuzumab vedotin) OR (Tisotumab vedotin) OR (Tivdak) OR (Trastuzumab duocarmazine) OR (U3-1402) OR (XMT-1536) OR (XMT-1592)).ti,ab,kf.
29. or/26-28
30. Targeted Therapy/ or exp Molecular Targeted Therapy/
31. (Targeted Therapy or Molecular Targeted Therapy).ti,ab,kf.
32. or/30-31
33. 6 and 29 and 32
34. 19 and 33

## Supplementary Method 3. Search strategy for Cochrane

1. MeSH descriptor: [Stomach Neoplasms] explode all trees
2. (Stomach Neoplasms):ti,ab,kw OR (((stomach):ti,ab,kw OR (gastric):ti,ab,kw) AND ((neoplas*):ti,ab,kw OR (cancer*):ti,ab,kw OR (carcino*):ti,ab,kw OR (adenocarcino*):ti,ab,kw OR (carcinoma):ti,ab,kw OR (malig*):ti,ab,kw OR (tumor*):ti,ab,kw OR (tumour*):ti,ab,kw))
3. #1 OR#2
4. MeSH descriptor: [Neoplasm Metastasis] explode all trees
5. (Metastases, Neoplasm):ti,ab,kw OR (Metastasis, Neoplasm):ti,ab,kw OR (Neoplasm Metastases):ti,ab,kw
6. (advanced):ti,ab,kw OR (stage III):ti,ab,kw OR (stage IV):ti,ab,kw OR (stage four) OR (unresectable):ti,ab,kw OR (inoperable):ti,ab,kw OR ((Metastase):ti,ab,kw OR (Metastases):ti,ab,kw OR (Metastasis):ti,ab,kw OR (metasta*):ti,ab,kw OR (advanced):ti,ab,kw) AND ((neoplas*):ti,ab,kw OR (cancer*):ti,ab,kw OR (carcino*):ti,ab,kw OR (adenocarcino*):ti,ab,kw OR (tumor):ti,ab,kw OR (tumors):ti,ab,kw OR (tumour):ti,ab,kw OR (tumours):ti,ab,kw OR (malig*):ti,ab,kw)
7. #4 OR #5 OR 6#
8. (Genes, erbB-2):ti,ab,kw OR (HER-2 Genes):ti,ab,kw OR (HER-2 Gene):ti,ab,kw OR (Genes, HER-2):ti,ab,kw OR (c-erbB-2 Genes):ti,ab,kw OR (c erbB 2 Proto Oncogenes):ti,ab,kw OR (erbB-2 Gene):ti,ab,kw OR (erbB-2 Genes):ti,ab,kw OR (erbB 2 Genes):ti,ab,kw OR (c-erbB-2 Gene):ti,ab,kw OR (c erbB 2 Genes):ti,ab,kw OR (c-erbB-2 Proto-Oncogene):ti,ab,kw OR (c-erbB-2 Proto-Oncogenes):ti,ab,kw
9. (Receptor, ErbB-2):ti,ab,kw OR (Proto-Oncogene Protein HER-2):ti,ab,kw OR (Oncogene Protein HER-2):ti,ab,kw OR (Oncogene Protein HER 2):ti,ab,kw OR (HER 2 Proto Oncogene Protein):ti,ab,kw OR (Proto Oncogene Protein HER 2):ti,ab,kw OR (HER-2 Proto-Oncogene Protein):ti,ab,kw OR (Proto-Oncogene Protein, HER-2):ti,ab,kw OR (erbB-2 Proto-Oncogene Protein):ti,ab,kw OR (erbB-2 Receptors):ti,ab,kw OR (erbB-2 Receptor Protein-Tyrosine Kinase):ti,ab,kw OR (ErbB-2 Receptor):ti,ab,kw OR (erbB 2 Receptor Protein Tyrosine Kinase):ti,ab,kw OR (c erbB 2 Protein):ti,ab,kw OR (Proto Oncogene Proteins c erbB 2):ti,ab,kw OR (erbB 2 Proto Oncogene Protein):ti,ab,kw OR (c-erbB-2 Protein):ti,ab,kw OR (Receptors, erbB-2):ti,ab,kw OR (Proto-Oncogene Protein, erbB-2):ti,ab,kw OR (Proto-Oncogene Proteins c-erbB-2):ti,ab,kw
10. #8 OR #9
11. MeSH descriptor: [Immunoconjugates] explode all trees
12. (ADC) OR (ADCs) OR (Conjugates, Antibody-Drug):ti,ab,kw OR (Antibody-Drug Conjugate):ti,ab,kw OR (Antibody Drug Conjugate):ti,ab,kw OR (Conjugate, Antibody Drug):ti,ab,kw OR (Conjugate, Antibody-Drug):ti,ab,kw OR (Antibody Drug Conjugates):ti,ab,kw OR (Antibody-Drug Conjugates):ti,ab,kw OR (Drug Conjugate, Antibody):ti,ab,kw
13. (Brentuximab Vedotin):ti,ab,kw OR (Adcetris):ti,ab,kw OR (SGN-35):ti,ab,kw OR (Belantamab Mafodotin):ti,ab,kw OR (Blenrep):ti,ab,kw OR (GSK2857916):ti,ab,kw OR (Cetuximab Saratolacan):ti,ab,kw OR (Akalux):ti,ab,kw OR (Enfortumab Vedotin):ti,ab,kw OR (Padcev):ti,ab,kw OR (ASG-22ME):ti,ab,kw OR (Gemtuzumab Ozogamicin):ti,ab,kw OR (Mylotarg):ti,ab,kw OR (CMA-676):ti,ab,kw OR (Elahere):ti,ab,kw OR (Inotuzumab Ozogamicin):ti,ab,kw OR (Besponsa):ti,ab,kw OR (CMC-544):ti,ab,kw OR (Moxetumomab Pasudotox):ti,ab,kw OR (Lumoxiti):ti,ab,kw OR (Polatuzumab Vedotin):ti,ab,kw OR (Polivy):ti,ab,kw OR (Sacituzumab Govitecan):ti,ab,kw OR (Trodelvy):ti,ab,kw OR (IMMU-132):ti,ab,kw OR (Trastuzumab Deruxtecan):ti,ab,kw OR (Enhertu):ti,ab,kw OR (DS-8201):ti,ab,kw OR (Trastuzumab Emtansine):ti,ab,kw OR (Kadcyla):ti,ab,kw OR (TDM-1):ti,ab,kw OR ("1959-sss-DM3":ti,ab,kw) OR (A166):ti,ab,kw OR (ABBV-085):ti,ab,kw OR (ABBV-321):ti,ab,kw OR (ABBV-399):ti,ab,kw OR (ADCT-301):ti,ab,kw OR (ADCT-402):ti,ab,kw OR ("A-dmDT390-bisFv":ti,ab,kw) OR (Anetumab ravtansine):ti,ab,kw OR (ARX788):ti,ab,kw OR (ATOR-1015):ti,ab,kw OR (B003):ti,ab,kw OR (BAT8001):ti,ab,kw OR ("BAY 94-9343":ti,ab,kw) OR (BAY-943):ti,ab,kw OR (BMS-986148):ti,ab,kw OR (BT-062):ti,ab,kw OR (BT1718):ti,ab,kw OR (BT5528):ti,ab,kw OR (Camidanlumab tesirine):ti,ab,kw OR (cofetuzumab pelidotin):ti,ab,kw OR (CX-2009):ti,ab,kw OR (CX-2029):ti,ab,kw OR (Datopotamab deruxtecan):ti,ab,kw OR (depatuxizumab mafodotin):ti,ab,kw OR (disitamab vedotin):ti,ab,kw OR (Dolaflexin):ti,ab,kw OR (DS-1062a):ti,ab,kw OR (DS-6157a):ti,ab,kw OR (Enapotamab vedotin):ti,ab,kw OR ("F0002-ADC":ti,ab,kw) OR (HuMax-TF-ADC):ti,ab,kw OR (IMGN-853):ti,ab,kw OR (Indatuximab ravtansine):ti,ab,kw OR (Ladiratuzumab vedotin):ti,ab,kw OR (L-DOS47):ti,ab,kw OR (Loncastuximab tesirine):ti,ab,kw OR (Lorvotuzumab Mertansine):ti,ab,kw OR (MEDI2228):ti,ab,kw OR (MEN1309):ti,ab,kw OR (MGC018):ti,ab,kw OR (Mirvetuximab soravtansine):ti,ab,kw OR (Mitazalimab):ti,ab,kw OR (MORAb-202):ti,ab,kw OR (Naratuximab emtansine):ti,ab,kw OR (OBI-999):ti,ab,kw OR (Oportuzumab Monatox):ti,ab,kw OR (patritumab deruxtecan):ti,ab,kw OR (PF-06647020):ti,ab,kw OR (PF-06804103):ti,ab,kw OR (PSMA-ADC):ti,ab,kw OR ("RC48-ADC":ti,ab,kw) OR (SAR408701):ti,ab,kw OR (Serclutamab talirine):ti,ab,kw OR (SGN-LIV1A):ti,ab,kw OR (SHR-A1403):ti,ab,kw OR (STRO-001):ti,ab,kw OR (SYD985):ti,ab,kw OR (TAK-164):ti,ab,kw OR (telisotuzumab vedotin):ti,ab,kw OR (Tisotumab vedotin):ti,ab,kw OR (Tivdak):ti,ab,kw OR (Trastuzumab duocarmazine):ti,ab,kw OR ("U3-1402":ti,ab,kw) OR (XMT-1536):ti,ab,kw OR (XMT-1592):ti,ab,kw
14. #11 OR #12 OR #13
15. #3 AND #7 AND #10 AND #14

## Supplementary Method 4. Search strategy for Scopus

TITLE-ABS-KEY(("stomach neoplasm*") OR ((stomach OR gastric) AND (neoplas* OR cancer* OR carcino* OR adenocarcino* OR tumor OR tumors OR tumour OR tumours OR malig*)))

AND

TITLE-ABS-KEY(("Neoplasm Metastasis" OR "Neoplasm Metastases") OR ((metastases OR metastasis OR metasta* OR advanced) AND (neoplas* OR cancer* OR carcino* OR adenocarcino* OR tumor OR tumors OR tumour OR tumours OR malig*)))

AND

(HER-2) OR (HER2) OR (ERBB2) OR (erbB-2) OR (Genes, erbB-2) OR (HER-2 Genes) OR (HER-2 Gene) OR (Genes, HER-2) OR (c-erbB-2 Genes) OR (c erbB 2 Proto Oncogenes) OR (erbB-2 Gene) OR (erbB-2 Genes) OR (erbB 2 Genes) OR (c-erbB-2 Gene) OR (c erbB 2 Genes) OR (c-erbB-2 Proto-Oncogene) OR (c-erbB-2 Proto-Oncogenes) OR (Receptor, ErbB-2) OR (Proto-Oncogene Protein HER-2) OR (Oncogene Protein HER-2) OR (Oncogene Protein HER 2) OR (HER 2 Proto Oncogene Protein) OR (Proto Oncogene Protein HER 2) OR (HER-2 Proto-Oncogene Protein) OR (Proto-Oncogene Protein, HER-2) OR (erbB-2 Proto-Oncogene Protein) OR (erbB-2 Receptors) OR (erbB-2 Receptor Protein-Tyrosine Kinase) OR (ErbB-2 Receptor) OR (erbB 2 Receptor Protein Tyrosine Kinase) OR (c erbB 2 Protein) OR (Proto Oncogene Proteins c erbB 2) OR (erbB 2 Proto Oncogene Protein) OR (c-erbB-2 Protein) OR (Receptors, erbB-2) OR (Proto-Oncogene Protein, erbB-2) OR (Proto-Oncogene Proteins c-erbB-2)

AND

(ADC) OR (ADCs) OR (Conjugates, Antibody-Drug) OR (Antibody-Drug Conjugate) OR (Antibody Drug Conjugate) OR (Conjugate, Antibody Drug) OR (Conjugate, Antibody-Drug) OR (Antibody Drug Conjugates) OR (Antibody-Drug Conjugates) OR (Drug Conjugate, Antibody) OR (Brentuximab Vedotin) OR (Adcetris) OR (SGN-35) OR (Belantamab Mafodotin) OR (Blenrep) OR (GSK2857916) OR (Cetuximab Saratolacan) OR (Akalux) OR (Enfortumab Vedotin) OR (Padcev) OR (ASG-22ME) OR (Gemtuzumab Ozogamicin) OR (Mylotarg) OR (CMA-676) OR (Elahere) OR (Inotuzumab Ozogamicin) OR (Besponsa) OR (CMC-544) OR (Moxetumomab Pasudotox) OR (Lumoxiti) OR (Polatuzumab Vedotin) OR (Polivy) OR (Sacituzumab Govitecan) OR (Trodelvy) OR (IMMU-132) OR (Trastuzumab Deruxtecan) OR (Enhertu) OR (DS-8201) OR (Trastuzumab Emtansine) OR (Kadcyla) OR (TDM-1) OR ("1959-sss-DM3") OR (A166) OR (ABBV-085) OR (ABBV-321) OR (ABBV-399) OR (ADCT-301) OR (ADCT-402) OR ("A-dmDT390-bisFv") OR (Anetumab ravtansine) OR (ARX788) OR (ATOR-1015) OR (B003) OR (BAT8001) OR ("BAY 94-9343") OR (BAY-943) OR (BMS-986148) OR (BT-062) OR (BT1718) OR (BT5528) OR (Camidanlumab tesirine) OR (cofetuzumab pelidotin) OR (CX-2009) OR (CX-2029) OR (Datopotamab deruxtecan) OR (depatuxizumab mafodotin) OR (disitamab vedotin) OR (Dolaflexin) OR (DS-1062a) OR (DS-6157a) OR (Enapotamab vedotin) OR ("F0002-ADC") OR (HuMax-TF-ADC) OR (IMGN-853) OR (Indatuximab ravtansine) OR (Ladiratuzumab vedotin) OR (L-DOS47) OR (Loncastuximab tesirine) OR (Lorvotuzumab Mertansine) OR (MEDI2228) OR (MEN1309) OR (MGC018) OR (Mirvetuximab soravtansine) OR (Mitazalimab) OR (MORAb-202) OR (Naratuximab emtansine) OR (OBI-999) OR (Oportuzumab Monatox) OR (patritumab deruxtecan) OR (PF-06647020) OR (PF-06804103) OR (PSMA-ADC) OR ("RC48-ADC") OR (SAR408701) OR (Serclutamab talirine) OR (SGN-LIV1A) OR (SHR-A1403) OR (STRO-001) OR (SYD985) OR (TAK-164) OR (telisotuzumab vedotin) OR (Tisotumab vedotin) OR (Tivdak) OR (Trastuzumab duocarmazine) OR ("U3-1402") OR (XMT-1536) OR (XMT-1592)

AND

(Clinical trial) OR (Trial)

AND NOT

(Animals) AND NOT (Humans)

Supplementary material – Tables

## Supplementary Table S1. Quality assessment of the included publications, based on the ROBINS-I tool (low, moderate, serious, critical, no information)

| Clinical trial | Types of bias | | | | | | | Overall score |
| --- | --- | --- | --- | --- | --- | --- | --- | --- |
|  | Bias  due to  confounding | Bias in  selection of  participants | Bias in  classification of  interventions | Bias due to  deviations from intended  interventions | Bias  due to  missing data | Bias in  measurement  of outcomes | Bias in  selection of  the reported  result |  |
| DS8201-A-J101 | Moderate | Low | Low | Moderate | Low | Moderate | Low | Moderate |
| Zhang et al. (2024) | Moderate | Low | Low | Moderate | Low | Moderate | Low | Moderate |
| DS8201-A-U205 | Serious | Moderate | Low | Moderate | Low | Low | Low | Serious |
| Xu et al. (2021) | Moderate | Low | Low | Low | Moderate | Moderate | Low | Moderate |
| Pegram et al. (2021) | Moderate | Low | Low | Low | Low | Moderate | Low | Low |
| Banerji et al. (2019) | Serious | Moderate | Low | Moderate | Low | Low | Moderate | Serious |
| Zhang et al. (2022) | Moderate | Low | Low | Low | Moderate | Moderate | Low | Moderate |
| Meric-Bernstam et al. (2023) | Serious | Moderate | Low | Serious | Moderate | Moderate | Moderate | Serious |
| DG-06 | Moderate | Low | Low | Moderate | Moderate | Low | Low | Moderate |

## Supplementary Table S2. Quality assessment of the included publications, based on the RoB 2.0 tool (low, high, unclear risk of bias)


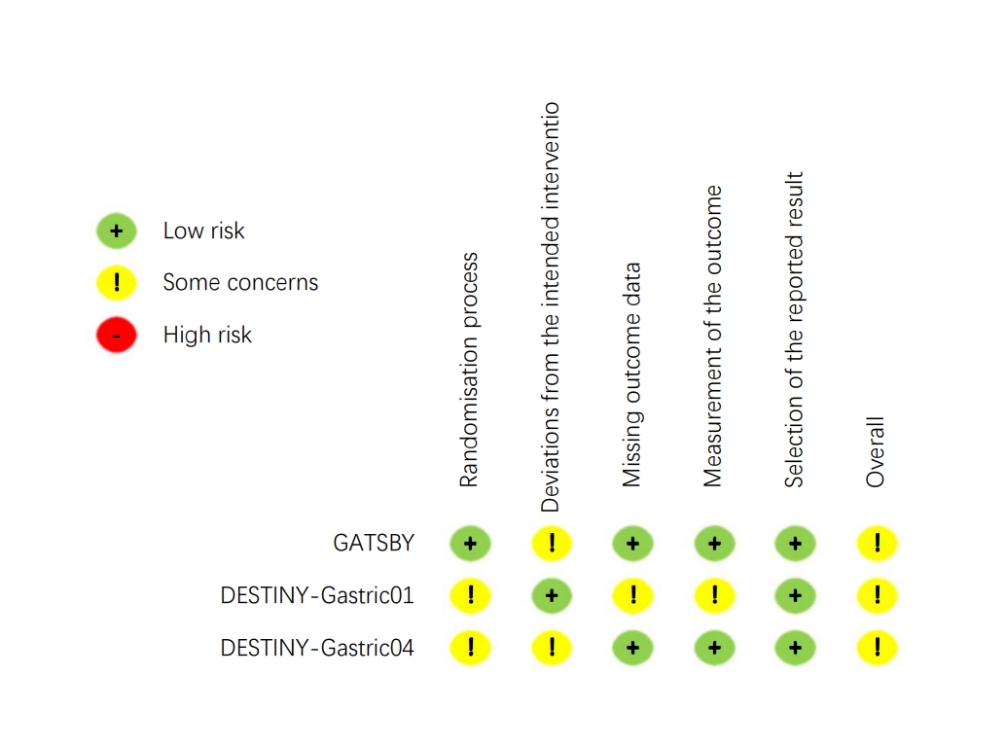


| Study ID | Types of bias | | | | | Overall Bias |
| --- | --- | --- | --- | --- | --- | --- |
|  | Randomization process | Deviations from intended interventions | Missing outcome data | Measurement of the outcome | Selection of the reported result |  |
| GATSBY | Low | Some concerns | Low | Low | Low | Some concerns |
| DESTINY-Gastric01 | Some concerns | Low | Some concerns | Some concerns | Low | Some concerns |
| DESTINY-Gastric04 | Some concerns | Some concerns | Low | Low | Low | Some concerns |

**
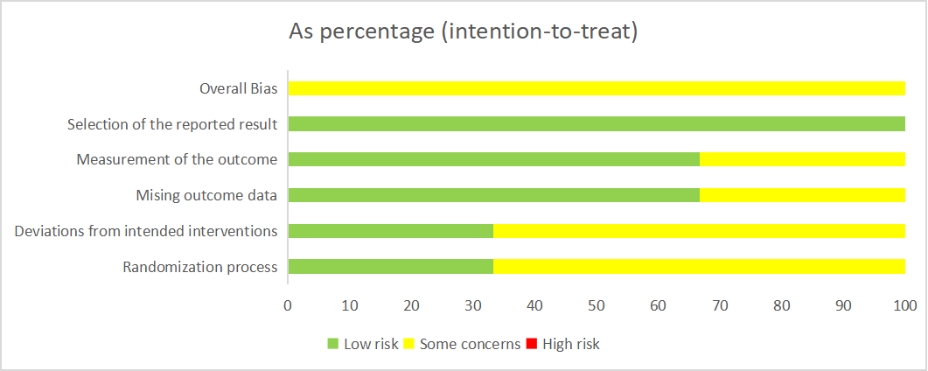
**

## Supplementary Table S3. Excluded Studies with Reasons

| DOI | Author (Year) | Apparent Eligibility | Primary Exclusion Reason |
| --- | --- | --- | --- |
| 10.1200/JCO.23.02005 | [Meric-Bernstam](https://pubmed.ncbi.nlm.nih.gov/?sort=pubdate&size=200&term=Meric-Bernstam+F&cauthor_id=37870536) et al. (2024) | Protocol match | Included population |
| 10.1007/s00761-020-00851-1 | Pamuk et al. (2021) | Study design | No detailed protocol |
| 10.1007/s10147-023-02422-x | Shitara et al. (2024) | Intervention match | Unmet criterion |
| 10.1158/2159-8290.CD-19-1014 | Tsurutani et al. (2020) | Study design | No outcome of interest |

Supplementary Table S4. All modifications to the protocol during its implementation

| Revision date | Revised content | Reasons for revision | Primary Exclusion Reason |
| --- | --- | --- | --- |
| 2025-08-07 | Expand the retrieval database to Scopus | Conduct a more comprehensive search for potential literature | More included studies |
| 2025-08-07 | Change the Review team members | Personnel changes occur | Optimize personnel arrangement |
| 2025-08-07 | Adjust publication bias | better analyze the data better | Optimize publication bias analysis |

## Supplementary Table S5. GRADE summary of findings table

| Certainty assessment | | | | | | | Impact | Certainty | | Importance |
| --- | --- | --- | --- | --- | --- | --- | --- | --- | --- | --- |
| № of studies | Study design | Risk of bias | Inconsistency | Indirectness | Imprecision | Other considerations |  |  |  |  |
| Overall Response Rate | | | | | | | | |  |  |
| 12 | randomised trials,  single-arm trials | serious^a^ | serious^b^ | not serious^b^ | serious^c^ | publication bias strongly suspected all plausible residual confounding would reduce the demonstrated effect dose response gradient^a,d^ | Funnel plot asymmetry, confirmed by Begg's test and Harbord's regression, indicated potential publication bias for ORR outcomes, suggesting underreporting of negative small-sample studies. Additionally, the inclusion of nine single-arm trials introduced risks of confounding and bias due to inherent non-randomization and small samples. To address limitations, analyses utilized a random-effects model; sensitivity analysis confirmed ORR effect size robustness regardless of any single study's exclusion. Subgroup analyses (GC patient proportion, prior lines, specific drug) failed to adequately explain heterogeneity. Furthermore, most drug subgroups comprised only single studies (excluding T-DXd), limiting cross-drug comparisons. | ⨁⨁◯◯ Low^a,b,c,d^ | | improtant |

CI: confidence interval

Explanations

^a^ 9/12 of these studies are single-arm studies or non-randomized controlled studies, which are deemed to have a risk of bias when assessed using the ROBINS-I tool.

^b^ Most single-arm studies have wide confidence intervals or large heterogeneity between studies (I ² > 50%).

^c^ ORR was selected as the primary endpoint because more single-arm studies were included. The absence of blinding inherent in non-randomized controlled trial designs, coupled with the inclusion of trials with small sample sizes, may have introduced unmeasured confounding biases.

^d^ RR > 2 for 1 RCT study

Supplementary material – Figure

## Supplementary Figure S1. Efficacy analysis of three randomized controlled trials; (A) Comparison of overall response rates; (B) Analysis of absolute risk differences; (C) Association between GC proportion and therapeutic efficacy.

**
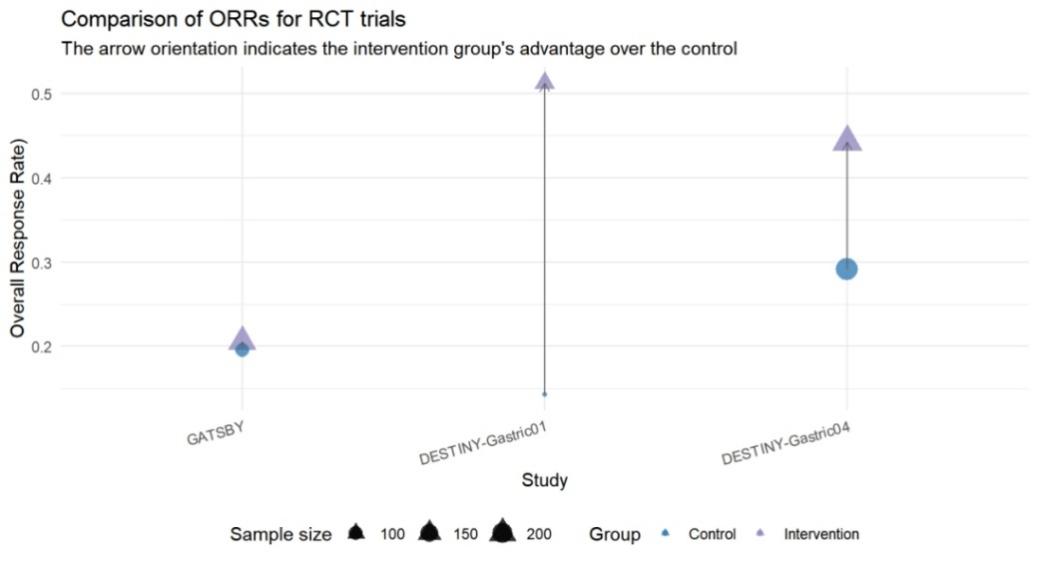
(A)**

**
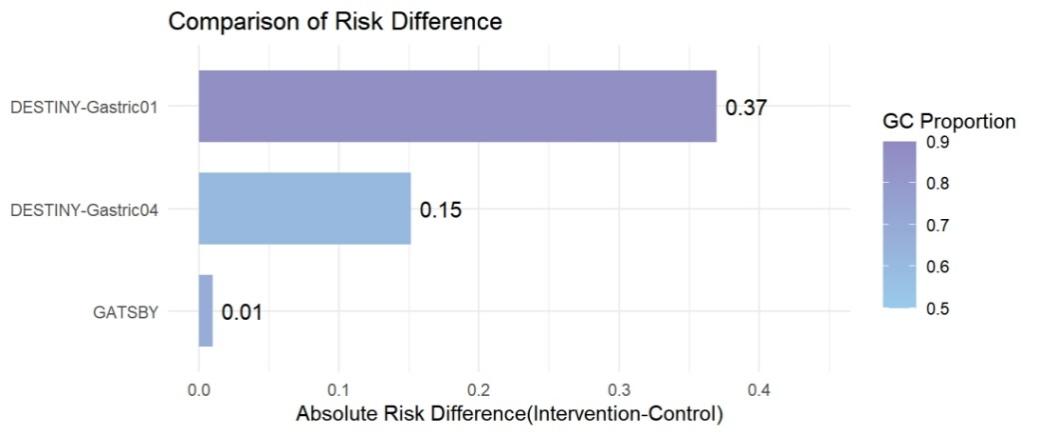
(B)**

**
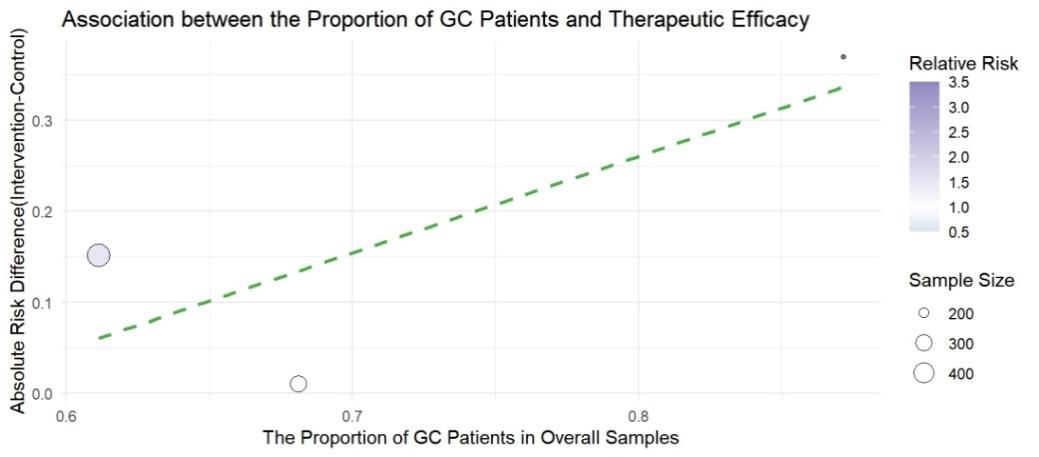
(C)**

## Supplementary Figure S2. Funnel plots of ORR from the included studies for the visual detection of systematic publication bias and small study effect.

|  |  |
| --- | --- |
|  | **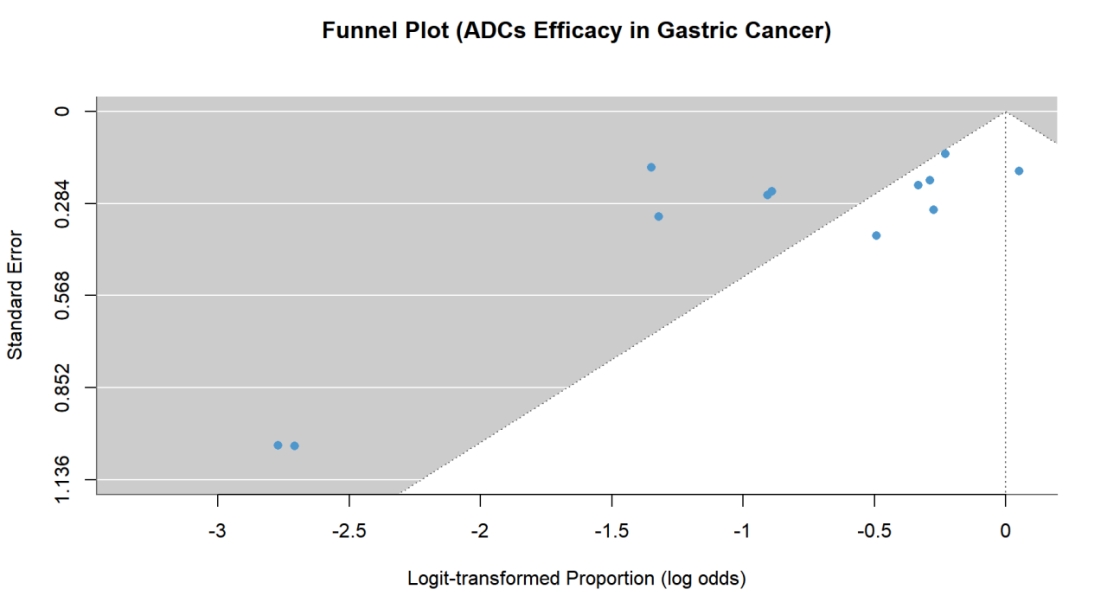** |

## Supplementary Figure S3. Harbord’s test of ORR from the included studies for the detection of publication.

|  |  |
| --- | --- |
|  | **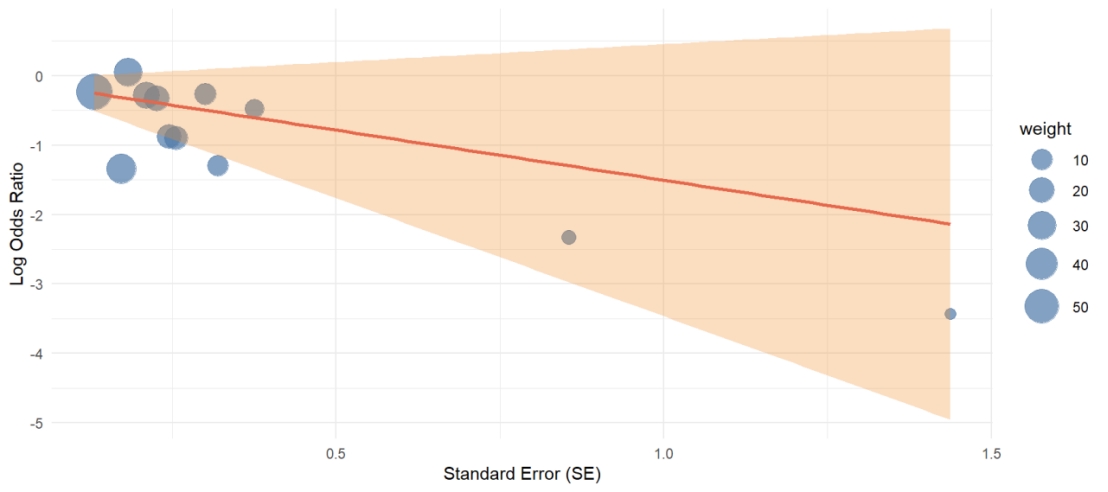** |

## Supplementary Figure S4. Sensitivity analysis of ORR in included trials for the robustness of findings to different aspects of the trials methodology.

**
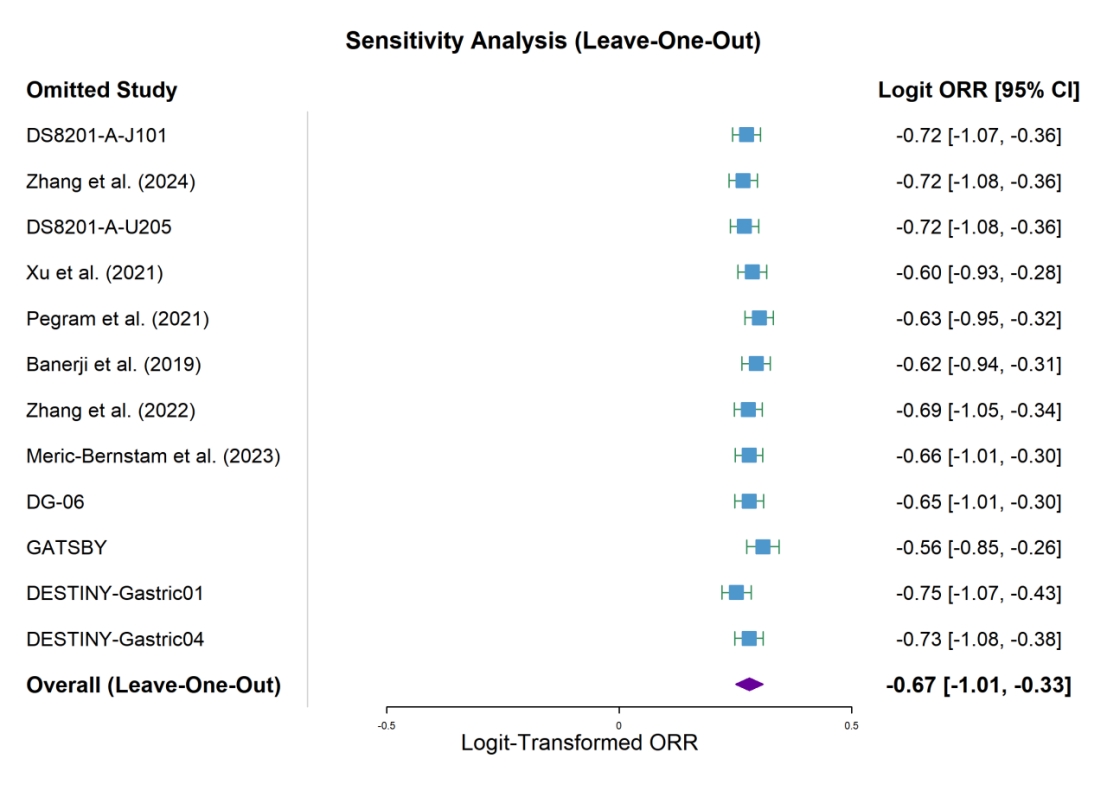
**

## Supplementary Figure S5. Subgroup analysis: ORR and 95% CI for HER2-positive GC or GEJ cancer patients treated with ADCs on basis of GC patients enrollment proportion (A), Prior therapeutic lines (B), Drug of intervention (C).

**
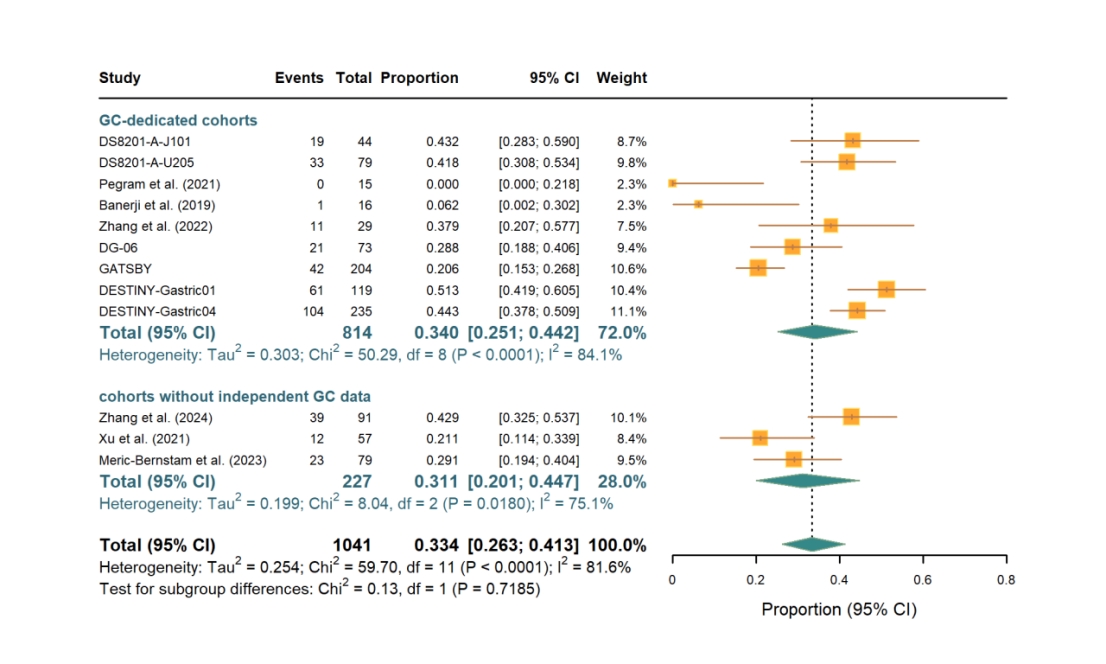
**

**(A)**

**
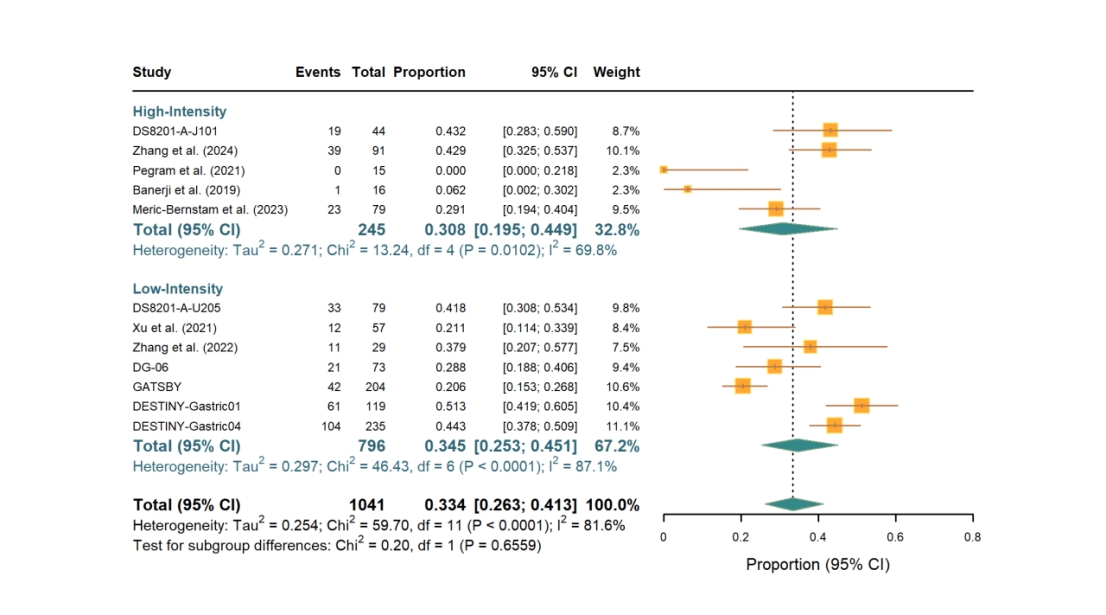
(B)**

**
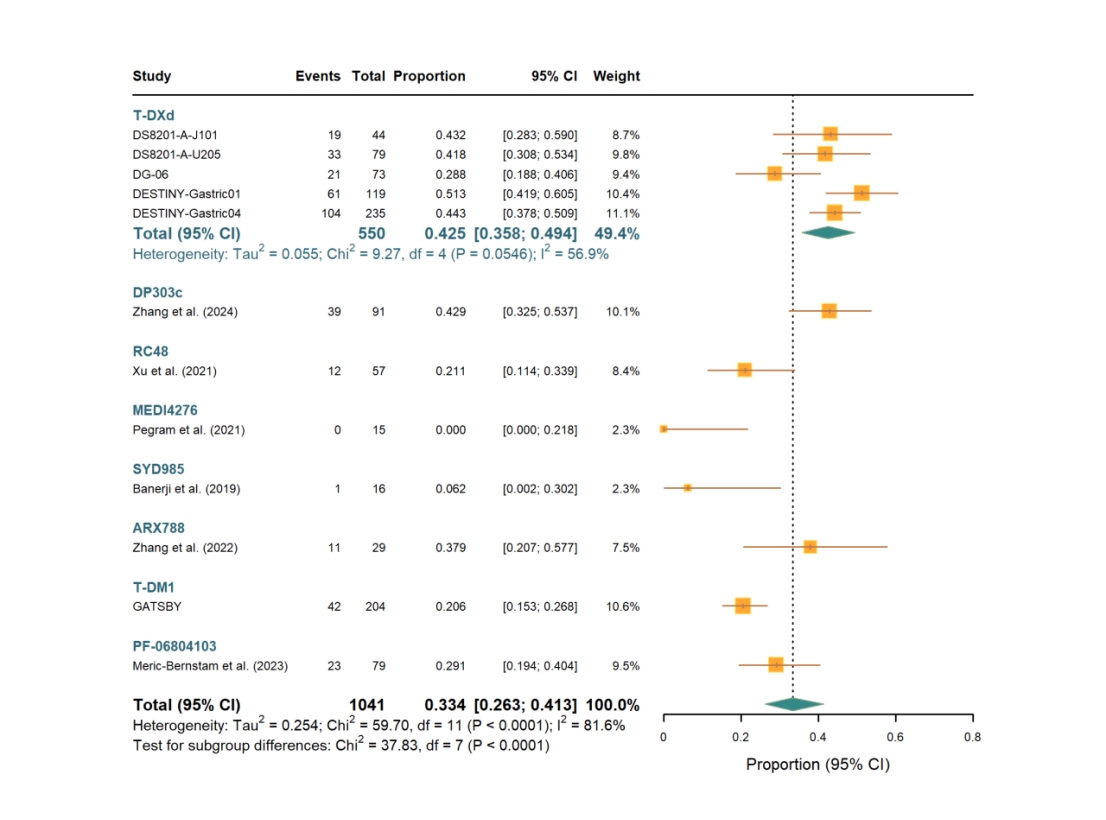
**

**(C)**

##
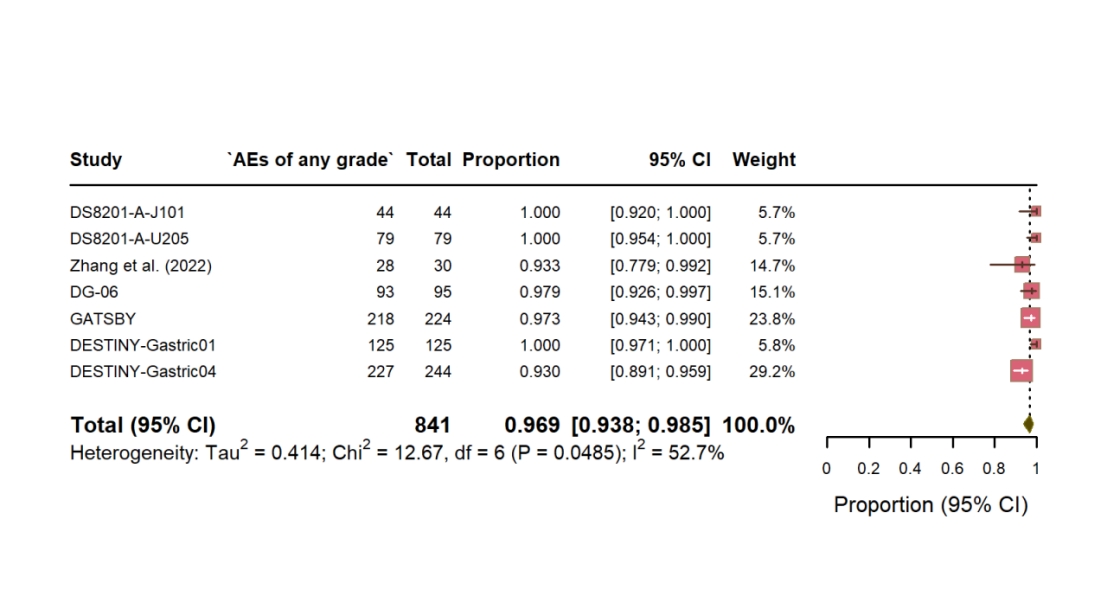
Supplementary Figure S6. Prevalence of: (A) AEs of any grade; (B) grade ≥ 3 AEs (B).


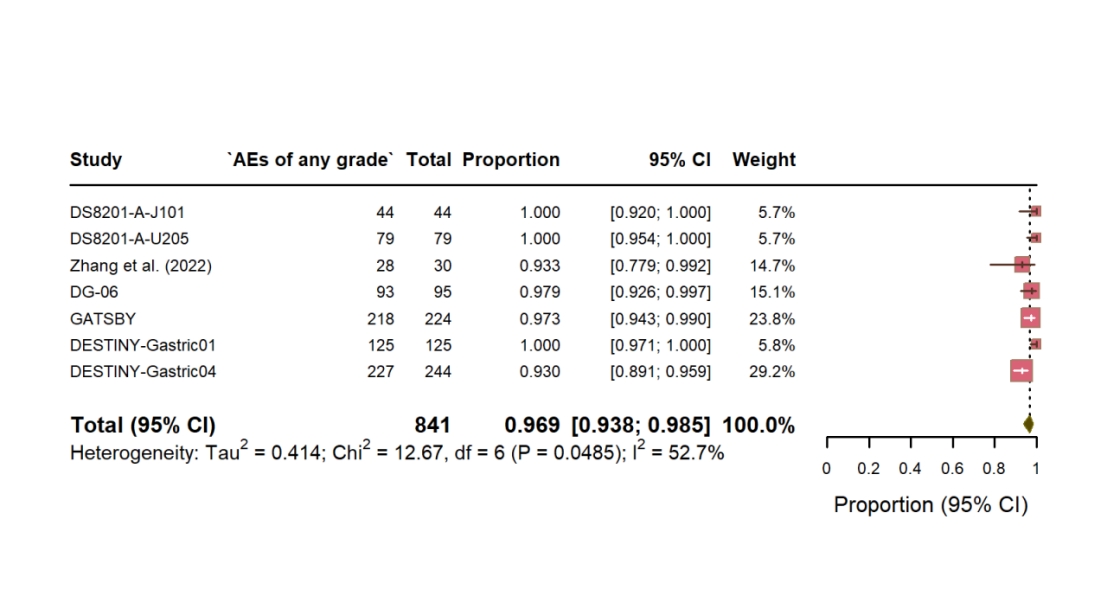


**(A)**

**
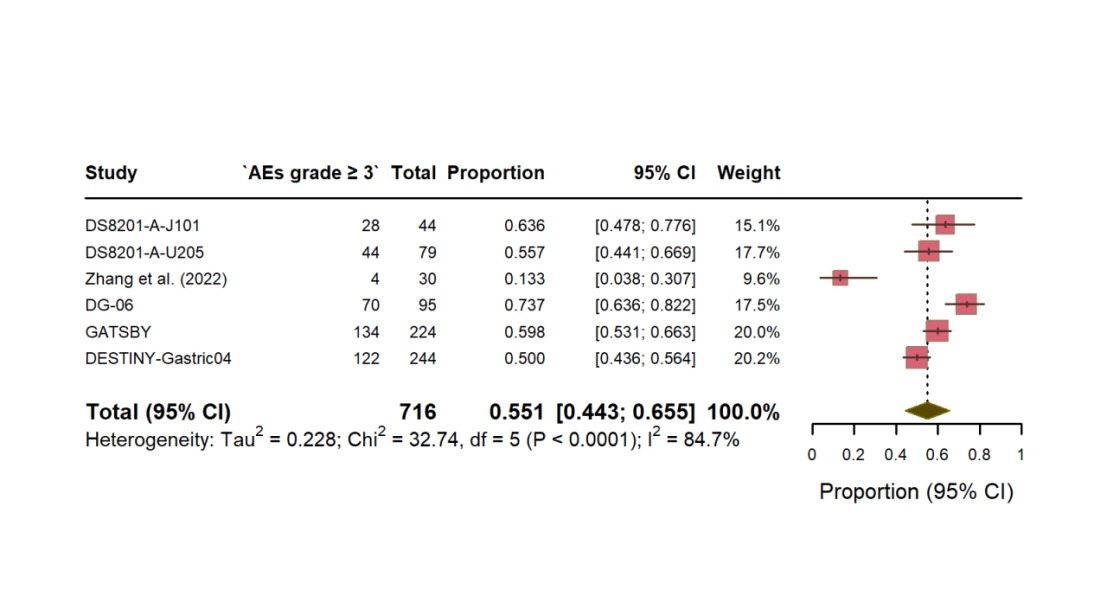
**

**
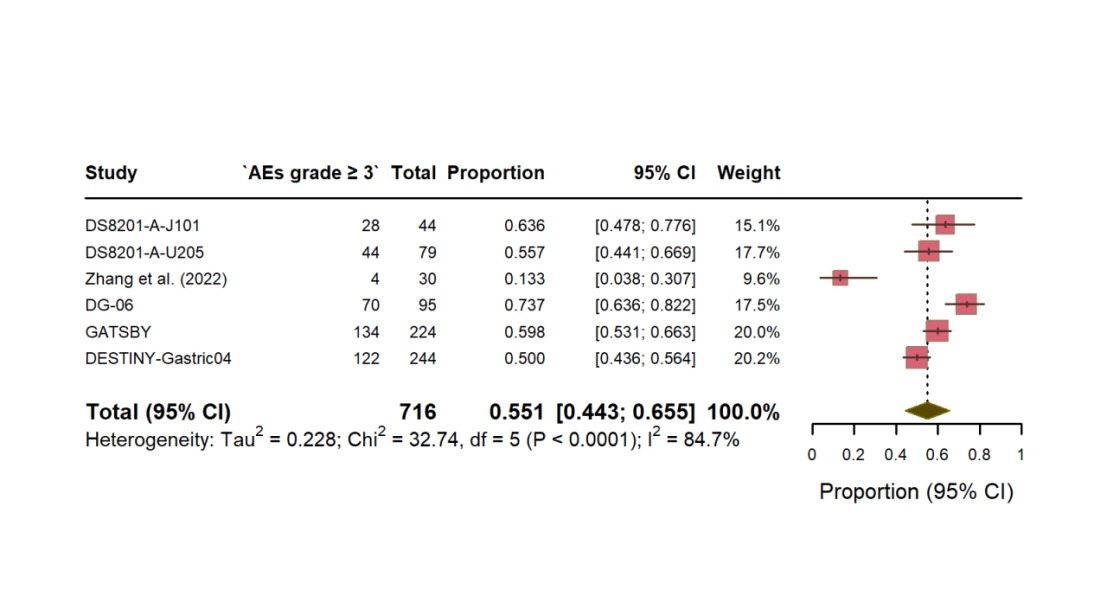
**

**(B)**

##
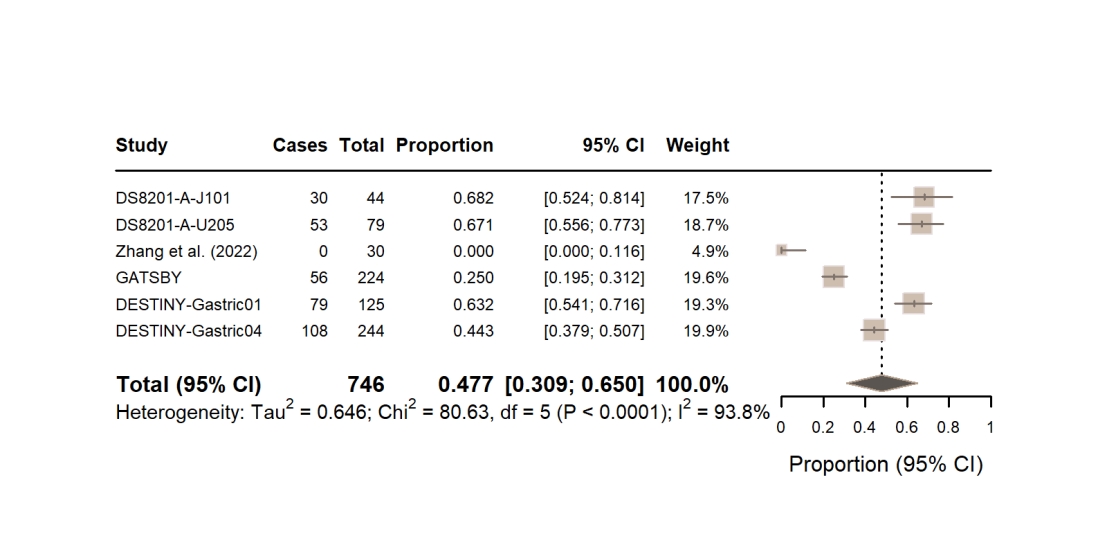
Supplementary Figure S7. Prevalence of the most common drug-related AEs : nausea (A), anaemia (B), and decreased appetite (C) of any grade; anaemia (D), neutropenia (E), leukopenia (F) of high-grade (≥ 3).

**(A)**

**
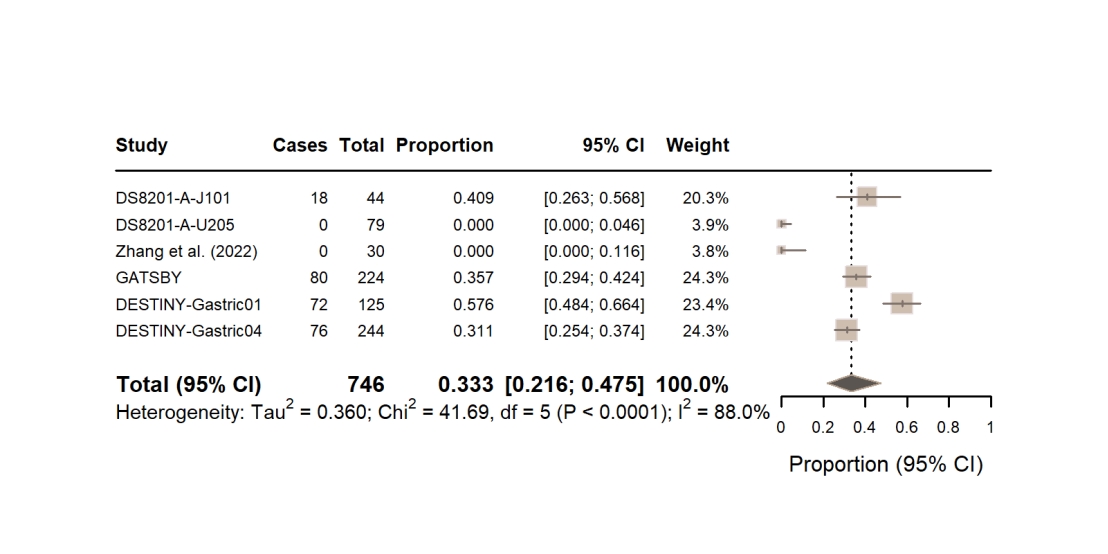
**

**(B)**

**
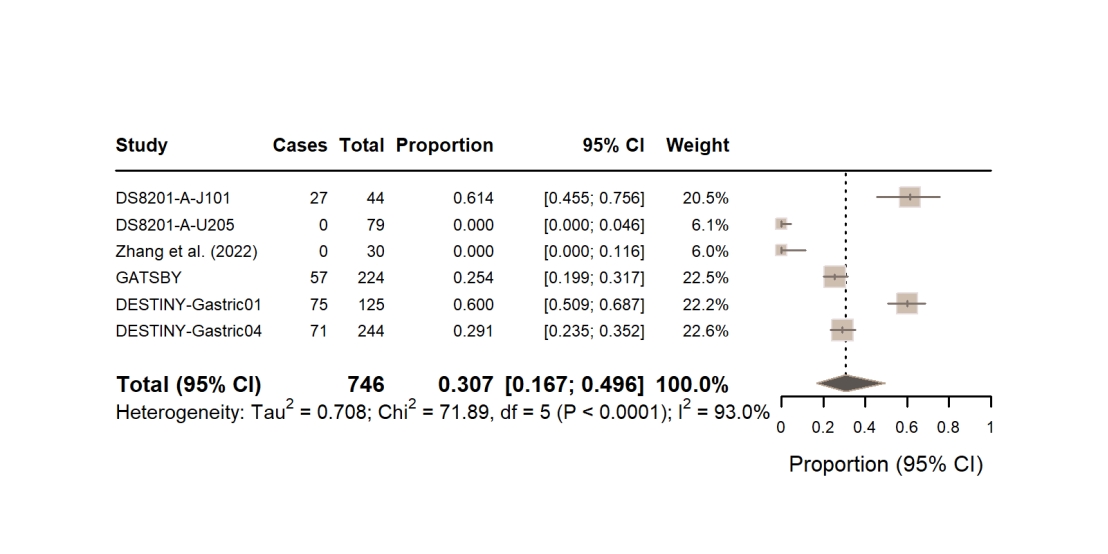
**

**(C)**

**
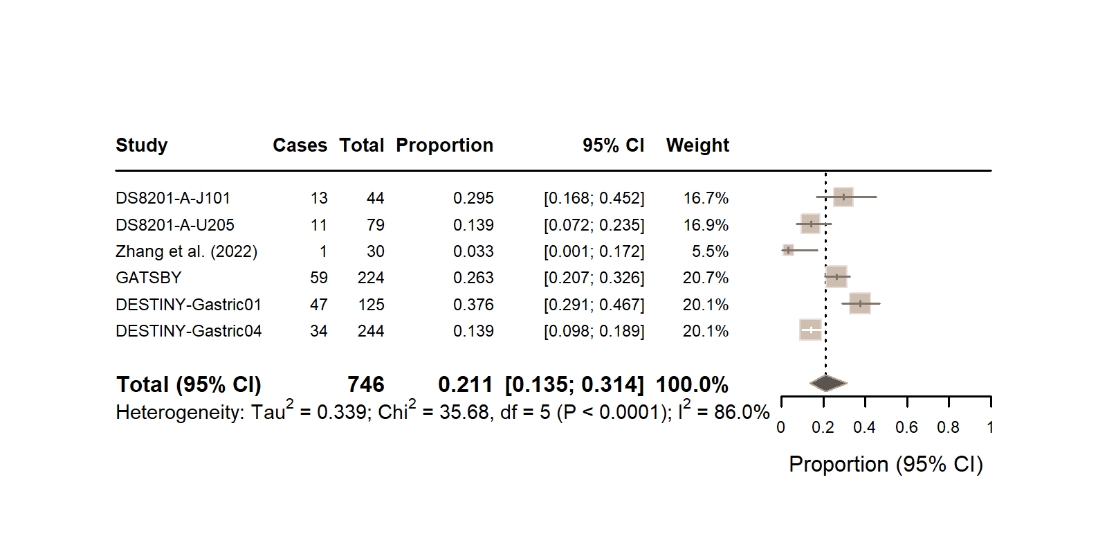
(D)**

**
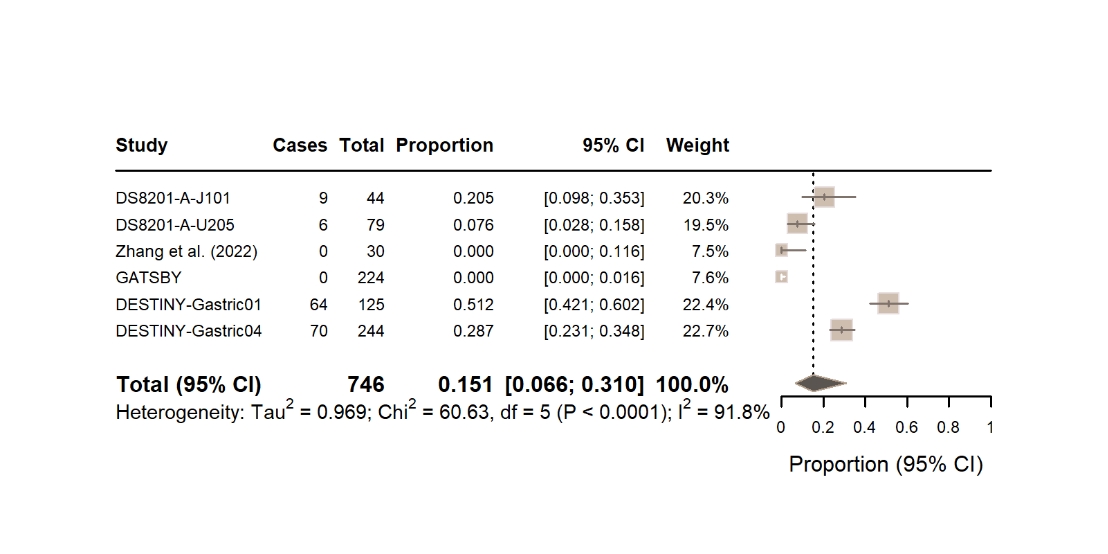
**

**(E)**

**
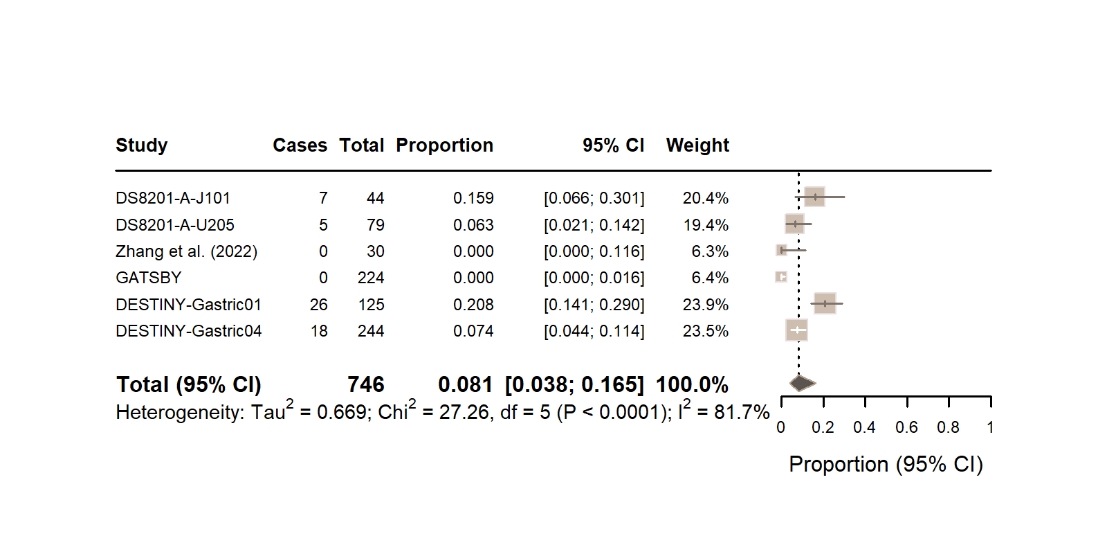
**

**(F)**

1. The First Clinical Medical School of Zhejiang Chinese Medical University, Hangzhou, Zhejiang, China. [↑](#footnote-ref-1)
2. The First Affiliated Hospital of Zhejiang Chinese Medical University, Hangzhou , Zhejiang, China.

   Correspondence: Shengliang Qiu. E-mail: shanmingruan@zcmu.edu.cn; [shengliang.qiu@zcmu.edu.cn](mailto:shengliang.qiu@zcmu.edu.cn)

   ^+^ These authors have contributed equally to this work and share first authorship [↑](#footnote-ref-2)
